# Supplementary material for: Hybrid Dihydropyrimidinones Targeting AKT Signaling: Antitumor Activity in Hormone-Dependent 2D and 3D Cancer Models
Source: Pharmaceutics. 2025 Nov 14;17(11):1470. doi: 10.3390/pharmaceutics17111470 (PMC12655361; doi:10.3390/pharmaceutics17111470)
Supplement: Supplementary file 1 [file pharmaceutics-17-01470-s001.zip › pharmaceutics-3934774-supplementary.pdf]

Hybrid Dihydropyrimidinones Targeting AKT Signaling: Antitumor Activity in Hormone-Dependent 2D and 3D Cancer Models

Amanda Helena Tejada[1], Samuel José Santos[2]; Gabriel Tofolli [1], Abu-Bakr A. Ariwoola[1]; Aryel José A. Bezerra[1]; Giulia Rodrigues Stringhetta[1]; Izabela Natalia Faria Gomes; Luciane Sussuchi da Silva[1]; Rui Manuel V. Reis [1,3,4]; Daniel D'Almeida Preto[5]; Dennis Russowsky [2*]; Renato José Silva-Oliveira [1,6*].

Supplementary Figures and Tables.

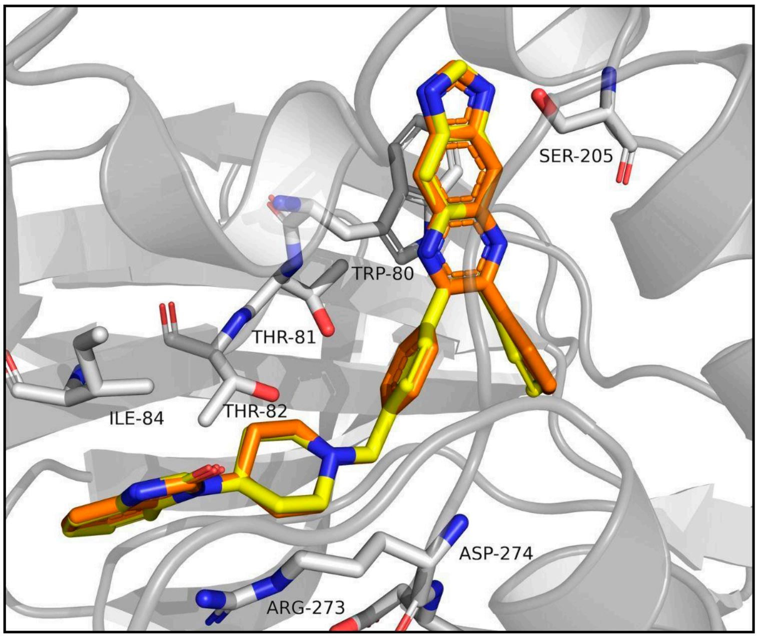

Supplementary Figure S1. Validation of the molecular docking protocol by redocking AVIII into AKT1. The co-crystallized ligand (PDB ID: 3O96, yellow sticks) is aligned with the top-ranked pose obtained from molecular docking of AVIII (orange sticks). Key interacting residues from AKT1 (gray cartoon) are shown as white sticks.

Supplementary Table S1. Chemical Structure of 22 of xanthene-dihydropyrimidinone hybrid compounds.

Name	Chemical Structure	Molecular weight (g mol^{-1})

**SJ022 (M1)**	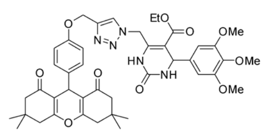	**795.88**
**SJ025 (M2)**	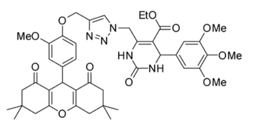	**825.90**
**SJ028 (M3)**	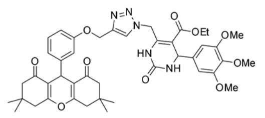	**795.88**
**SJ064 (M4)**	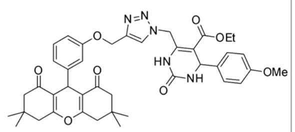	**735.83**
**SJ071 (M5)**	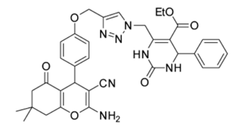	**649.70**
**SJ072 (M6)**	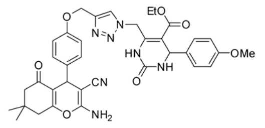	**679.72**
**SJ074 (M7)**	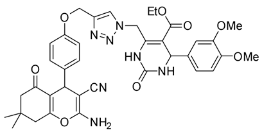	**709.75**
**SJ075 (M8)**	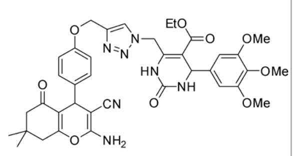	**739.77**
**SJ077 (M9)**	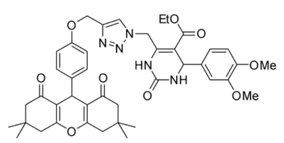	**765.85**

**SJ078 (M10)**	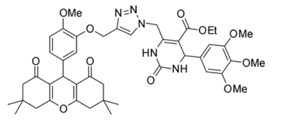	**825.90**
**SJ079 (M11)**	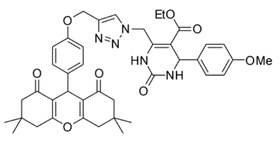	**735.83**
**SJ080 (M12)**	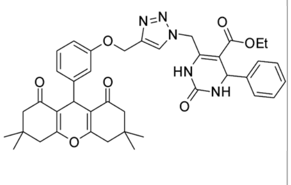	**705.80**
**SJ084 (M13)**	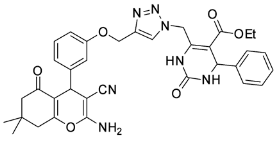	**649.70**
**SJ085 (M14)**	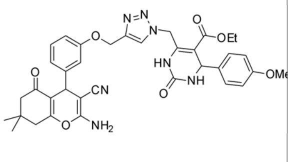	**679.72**
**SJ086 (M15)**	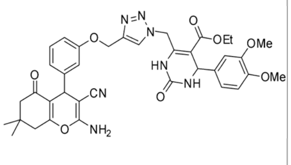	**709.75**
**SJ087 (M16)**	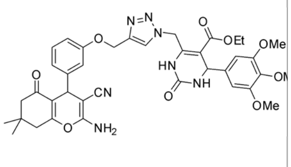	**739.77**
**SJ092 (M17)**	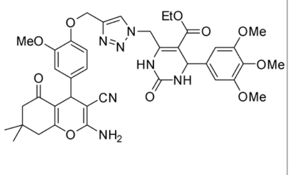	**769.80**
**SJ093 (M18)**	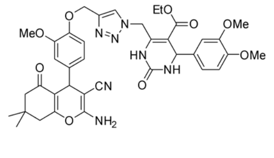	**739.77**

**SJ094 (M19)**	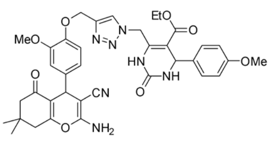	**709.75**
**SJ099 (M20)**	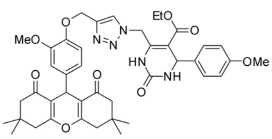	**765.85**
**SJ100 (M21)**	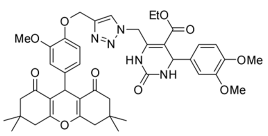	**795.88**
**SJ103 (M22)**	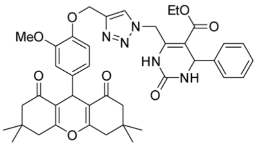	**735.83**

Supplementary Table S2. Lipinski's Rule of Five parameters for the molecules SJ028, SJ064, and SJ078. The table shows molecular weight (MW), number of hydrogen bond donors (HBD) and acceptors (HBA), predicted partition coefficient (Consensus Log P), and the number of Lipinski violations. These parameters are commonly used to estimate oral absorption and potential bioavailability of small molecules.

Molecule	Molecular Weight	H-bond acceptors (HBA)	H-bond donors (HBD)	Consensus Log P	Lipinski violations
SJ028	795.88	12	2	4.40	2
SJ064	735.82	10	2	4.40	2
SJ078	825.90	13	2	4.34	2

Supplementary Table S3. Selectivity indices (SI) of all compounds that passed the one-dose screening and were further evaluated in five-dose assays. The table presents SI values across different cancer cell lines, indicating the relative cytotoxicity of each compound toward tumor versus non-tumor cells. Higher SI values reflect greater selectivity for cancer cells, providing insight into the therapeutic potential of the compounds.

Molecule	HFF-1	PC-3	LNCaP	T-47D	MDA-MB-231	SKBR-3	SKOV-3	OVCAR-3
SJ022	NE	NE	>2.01	NE	NE	NE	NE	NE
SJ025	NE	NE	NE	NE	NE	NE	NE	NE
SJ028	NE	>3.92	>2.90	> 10.17	>8.88	NE	NE	>2.19
SJ064	NE	NE	NE	>6.64	>4.87	NE	NE	NE
SJ071	NE	NE	NE	>4.47	>3.49	NE	NE	NE
SJ072	NE	NE	NE	NE	NE	NE	NE	NE
SJ074	NE	NE	NE	NE	NE	NE	NE	NE
SJ075	NE	NE	NE	>4.35	NE	>2.12	NE	NE
SJ077	NE	NE	NE	NE	NE	NE	NE	NE
SJ078	NE	>2.34	NE	>2.24	>4.27	NE	NE	NE
SJ079	NE	NE	NE	NE	NE	NE	NE	NE
SJ080	NE	NE	NE	NE	>2.43	NE	NE	NE
SJ084	NE	NE	NE	>3.19	>2.08	NE	>2.02	NE
SJ085	NE	NE	NE	NE	NE	NE	NE	NE
SJ086	NE	NE	NE	NE	NE	NE	NE	NE
SJ087	NE	>2.14	NE	NE	NE	NE	NE	NE

SJ092	NE	NE	<2.31	NE	NE	NE	NE	NE
SJ093	NE	NE	NE	NE	NE	NE	NE	NE
SJ094	NE	NE	NE	>2.79	NE	NE	NE	NE
SJ099	NE	NE	NE	NE	NE	NE	NE	NE
SJ100	NE	NE	NE	NE	NE	NE	NE	NE
SJ103	NE	NE	NE	NE	NE	NE	NE	NE

NE: non-effective.

Supplementary Table S4. IC_{50} values (µM) of the hormonal therapies treatment (tamoxifen, fulvestrant and letrozole) in the T47D, LNCaP, and OVCAR-3 cell lines.

T-47D			LNCap			OVCAR-3		
**Tamoxifen**	**Fulv**	**Letrozole**	**Tamoxifen**	**Fulv**	**Letrozole**	**Tamoxifen**	**Fulv**	**Letrozole**
0.30µM	0.76µM	1.63µM	0.28µM	NE	NE	0.01µM	3.04µM	NE

Fulv.: fulvestrant; NE: non-effective.

Supplementary Table S5. Synergy analysis of hybrid compounds with hormonal therapies. Synergy scores obtained from analysis using the ZIP (Zero Interaction Potency), Loewe, and Bliss models on the SynergyFinder platform for combinations of the hybrid molecule with selected hormonal therapies in the T47D, OVCAR-3, and LNCaP cell lines. Scores were calculated based on the cell viability matrix generated by combined treatments at 0, IC_{25}, IC_{50}, and IC_{75} concentrations of each compound. The ZIP model estimates the expected response of a combination assuming no interaction between drugs, considering only the individual potency of each. Loewe and Bliss models provide complementary assessments of combination effects based on dose additivity and probabilistic interaction, respectively. Scores below –10 are interpreted as antagonistic, scores between –10 and +10 as additive, and scores above +10 indicate pharmacological synergy.

	Tamoxifen					Fulvestrant			Letrozole	
	T-47D		LNCap		OVCAR-3	T-47D		OVCAR-3	T-47D	
SCORES	SJ028	SJ064	SJ028	SJ078	SJ028	SJ028	SJ064	SJ028	SJ028	SJ064
ZIP	64.73	35.75	-2.10	-3.84	25.67	31.04	22.47	23.82	4.50	9.53
Bliss	64.58	35.70	-3.70	-6.27	24.29	30.56	21.99	23.46	5.08	9.14
Loewe	44.71	5.62	-23.80	-18.01	8.44	30.51	4.00	11.50	-14.45	3.70

Effect	Synergistic	Synergistic	Additive	Additive	Synergistic	Synergistic	Synergistic	Synergistic	Additive	Additive

Supplementary Table S6. Synergy analysis of hybrid compounds with pharmacological AKT inhibitor MK2206. Synergy scores obtained from analysis using the ZIP (Zero Interaction Potency), Loewe, and Bliss models on the SynergyFinder platform for combinations of the hybrid molecule with pharmacological AKT inhibitor MK2206 in the T47D, OVCAR-3, and LNCaP cell lines. Scores were calculated based on the cell viability matrix generated by combined treatments at 0, IC_{25}, IC_{50}, and IC_{75} concentrations of each compound. The ZIP model estimates the expected response of a combination assuming no interaction between drugs, considering only the individual potency of each. Loewe and Bliss models provide complementary assessments of combination effects based on dose additivity and probabilistic interaction, respectively. Scores below –10 are interpreted as antagonistic, scores between –10 and +10 as additive, and scores above +10 indicate pharmacological synergy.

SCORES	SJ028 + MK2206	SJ064 + MK2206	SJ028 + MK2206	SJ078 + MK2206	SJ028 + MK2206
**ZIP**	-37,56	-12,243	-46,842	-45,582	-17,406
**Bliss**	-36,038	-12,647	-45,96	-45,088	-16,572
**Loewe**	-27,258	-8,065	-30,834	-34,037	-12,817
**Effect**	Antagonistic	Antagonistic	Antagonistic	Antagonistic	Antagonistic

Supplementary Table S7. Molecular docking-estimated binding energies of the molecules to AKT1 allosteric pocket. Binding affinity energies of the control and the hybrid molecules in nine different binding conformations to the allosteric site of the AKT1 protein, obtained by molecular docking using AutoDock Vina v1.2.0. The energies are expressed in kcal/mol and represent an estimate of the stability of the molecules-AKT1 complexes. More negative values indicate higher binding affinity, suggesting more stable interactions between the molecule and the binding site. Generally, values below –7 kcal/mol are considered indicative of biologically relevant interactions, while affinities below –9 kcal/mol are associated with strong binding, potentially in the sub-micromolar range, which is desirable for bioactive molecules.

	VIII	SJ028	SJ064	SJ078
**Position**	**Affinity (kcal/mol)**	**Affinity (kcal/mol)**	**Affinity (kcal/mol)**	**Affinity (kcal/mol)**
**1**	-14.3	-12.3	-12.5	-11.1
**2**	-14.1	-12.2	-12.4	-11.0
**3**	-13.4	-12.2	-12.4	-10.5
**4**	-13.1	-12.0	-12.3	-10.2
**5**	-12.8	-11.8	-12.2	-10.1
**6**	-12.5	-11.7	-12.1	-10.0
**7**	-12.4	-11.6	-12.0	-10.0
**8**	-12.4	-11.4	-11.4	-9.9
**9**	-12.4	-11.3	-11.4	-9.9

Supplementary Table S8. IC_{50} values of resistant cancer cell lines treated with hormonal therapies and hybrid molecules. IC_{50} values (μM) of the resistant T47D, OVCAR-3, and LNCaP

cell lines treated with hormonal therapies (tamoxifen, fulvestrant, or letrozole) or with hybrid molecules derived from xanthene and pyran. The resistant cell lines exhibited IC_{50} values of at least 35 µM, confirming the development of acquired resistance. In contrast, the IC_{50} values for the hybrid drugs remained similar between parental and resistant cell lines, indicating that these compounds maintain their cytotoxic efficacy even in the context of hormonal resistance.

**T-47D** Resistant to tamoxifen (µM)		**T-47D** Resistant to fulvestrant (µM)		**T-47D** Resistant to letrozole (µM)		**Parental T-47D**	
**SJ028**	**SJ064**	**SJ028**	**SJ064**	**SJ028**	**SJ064**	**SJ028**	**SJ064**
4.36	5.15	4.37	5.72	4.51	5.81	3.44	5.27

**T-47D** Resistant to letrozole (µM)		**LNCap** Resistant to tamoxifen (µM)		**Parental LNCap** (µM)	
**SJ028**	**SJ064**	**SJ028**	**SJ078**	**SJ028**	**SJ078**
4.51	5.81	1.60	16.18	12.08	17.38

**OVCAR-3** Resistant to tamoxifen (µM)	**OVCAR-3** Resistant to fulvestrant (µM)	**Parental OVCAR-3**
**SJ028**	**SJ028**	**SJ028**
9.72	11.39	18.72

Preparation of Hybrid Compounds*

Xanthenes were prepared in one step through a pseudo-tricomponent reaction between dimedone (2 Equiv.) and appropriate oxy-propargyl aldehyde previously prepared from respective hydroxy aldehydes (scheme 1).

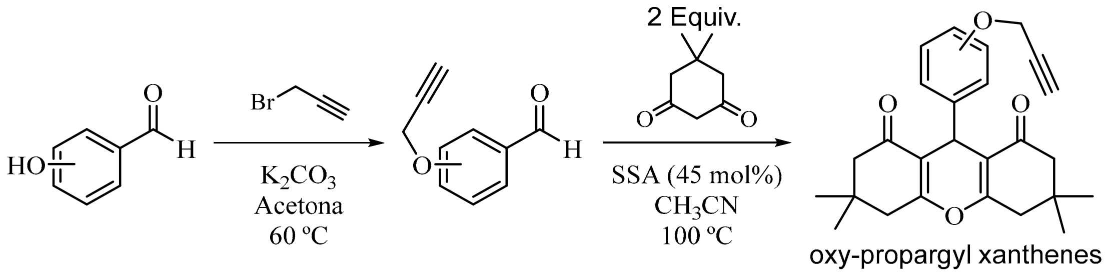

Scheme S1. Preparation od oxy-propargyl xanthenes

In turn, the pyrans were prepared in one step via a tricomponent reaction involving dimedone (1 equiv.), oxy-propargyl aldehyde (1 Equiv.), and malononitrile (1 Equiv., scheme 2).

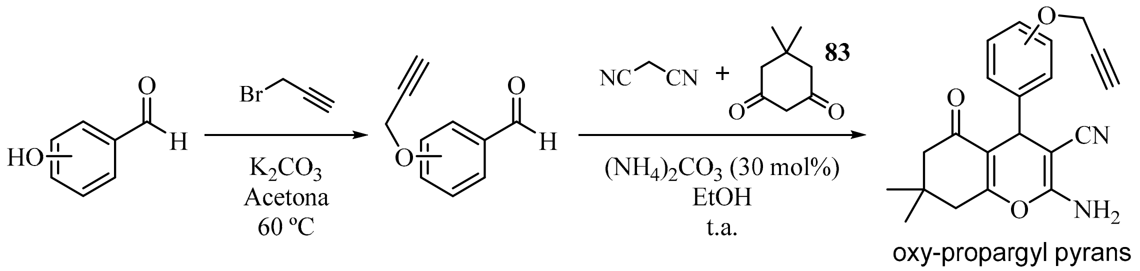

Scheme S2. Preparation of oxy-propargyl pyrans

The 6-azido dihydropyrimidinones were synthesized in two steps using the Biginelli tricomponent reaction with ethyl 4-chloro acetoacetate (1 equiv.), oxy-propargyl aldehyde (1 equiv.), and urea (1 equiv.). The resulting 6-chloro dihydropyrimidinone was converted to 6-azido dihydropyrimidinones by reacting with sodium azide, as shown in scheme 3.

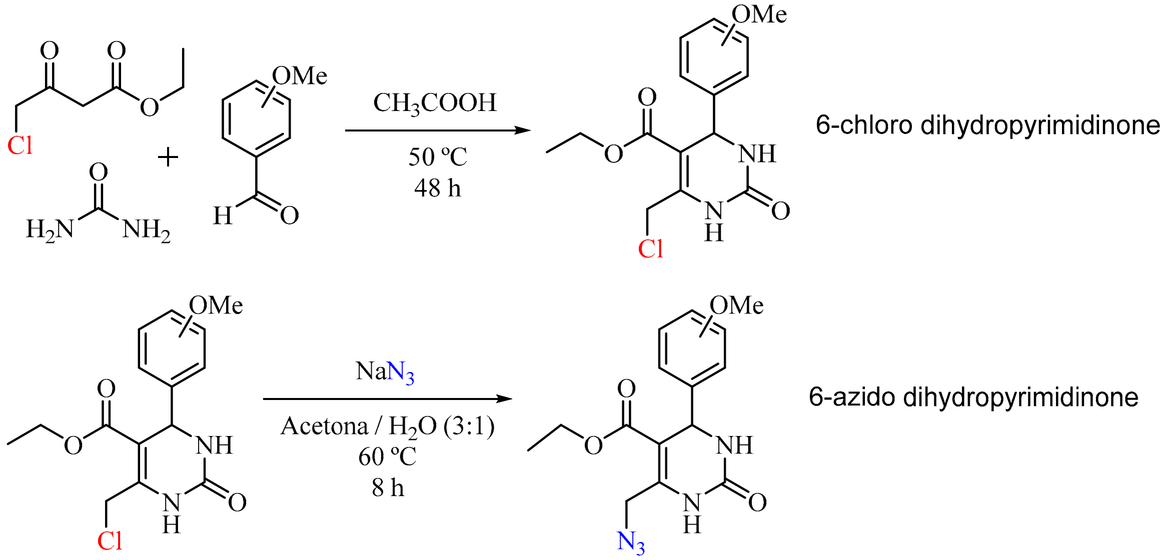

Scheme S3. Preparation of 6-azido dihydropyrimidinone

The *Click Reaction* of oxy-propargyl xanthenes or oxy-propargyl pyrans with 6-azido dihydropyrimidinones under copper(I) catalysis produces the hybrid compounds (scheme 4).

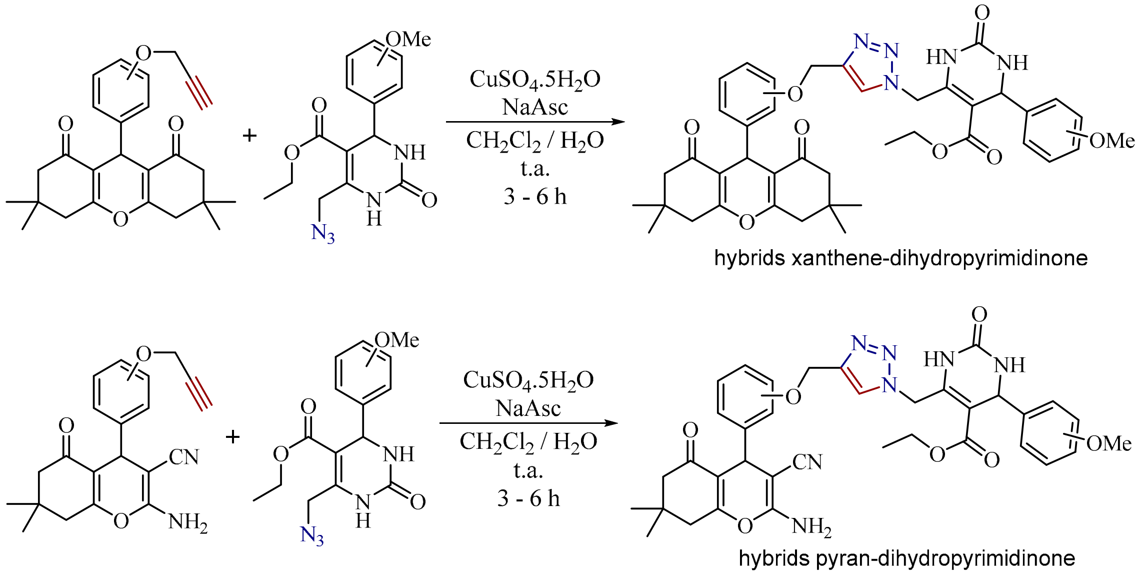

Scheme S4. Synthesis of xanthene-dihydropyrimidinone and pyran-dihydropyrimidinone hybrids

* For detailed information about the synthesis of hybrid compounds, please consult:

S. J. Santos, F. C. P. Rossatto, N. S. Jardim, D. S. A´vila, R. Ligabue-Braun, L. A. M. Fontoura, K. R. Zimmer, D. Russowsky, *Chromene-dihydropyrimidinone and xanthene-dihydropyrimidinone hybrids: design, synthesis, and antibacterial and antibiofilm activities. New J. Chem.* **2023**, *47*, 7500-7520.

^{1}H NMR, ^{13}C NMR & HRMS of Xanthene-Dihydropyrimidinone Hybrids

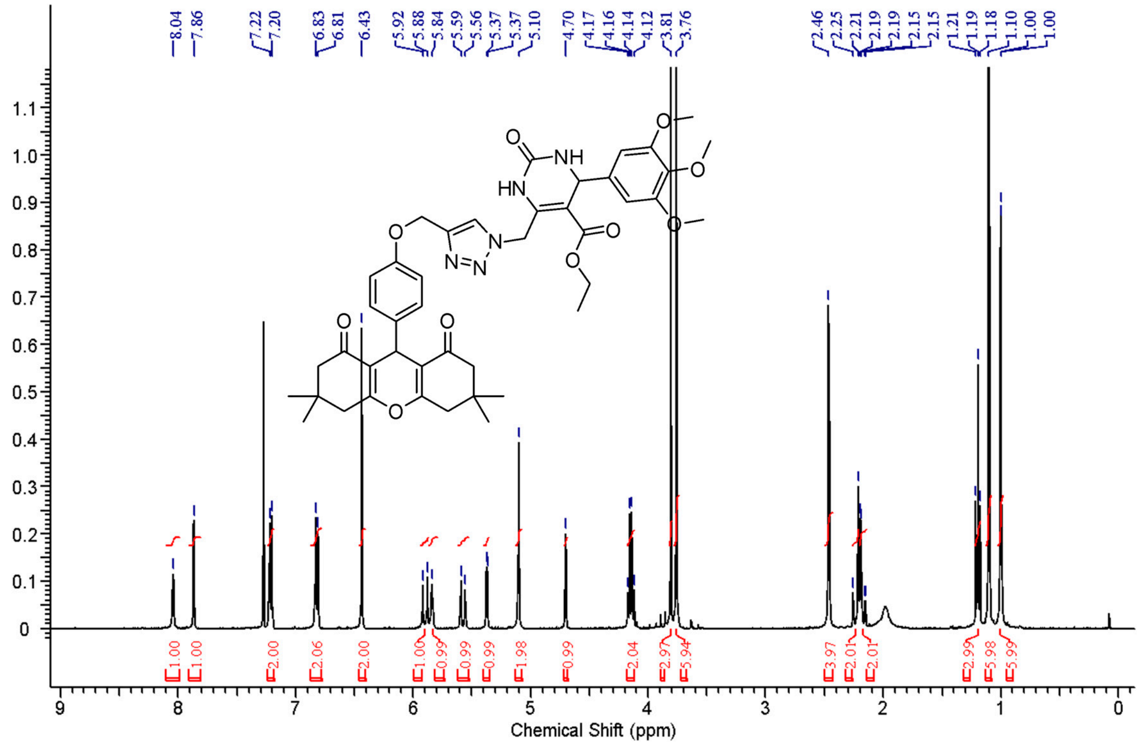

Figure S2. ^{1}H NMR (400 MHz, DMSO-*d*6) of Hybrid Compound **SJ022**

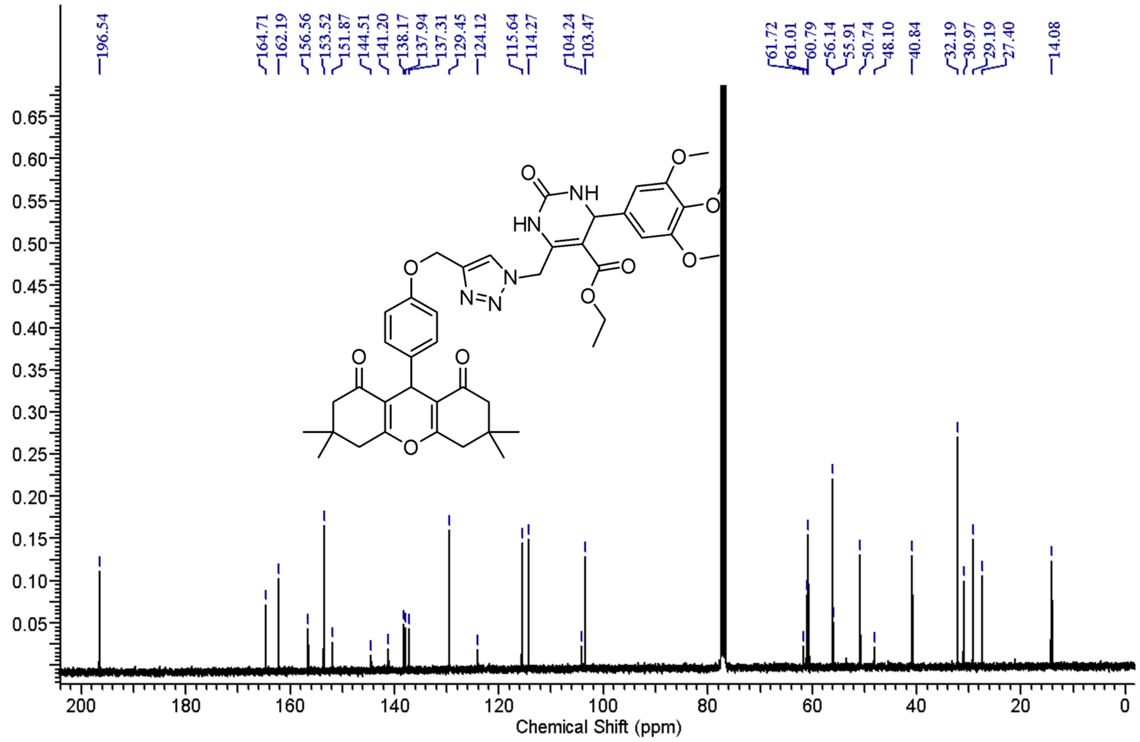

Figure S3: ^{13}C NMR (100 MHz, $CDCl_3$) of Hybrid Compound **SJ022**

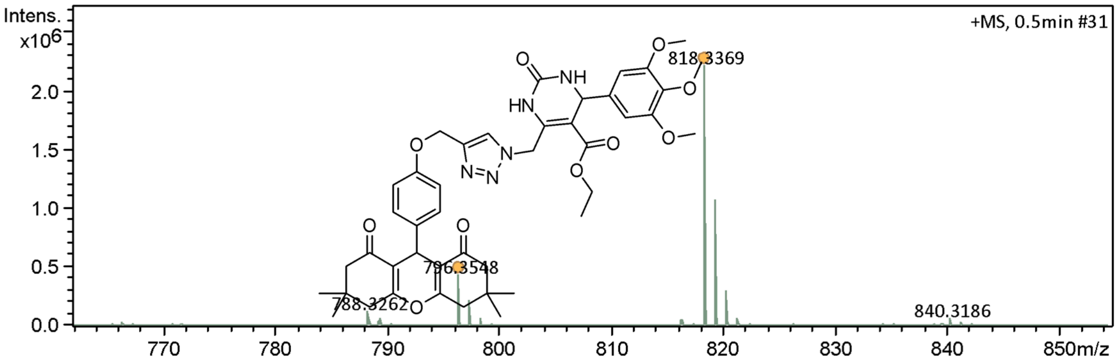

Meas. m/z	Ion Formula	m/z	err [ppm]	mSigma	rdb	e¯ Conf	N-Rule
796.3548	$C_{43}H_{50}N_5O_{10}$	796.3552	0.5	5.2	21.5	even	ok
818.3369	$C_{43}H_{49}N_5NaO_{10}$	818.3372	0.3	6.8	21.5	even	ok

Figure S4. HRMS of Hybrid Compound **SJ022**

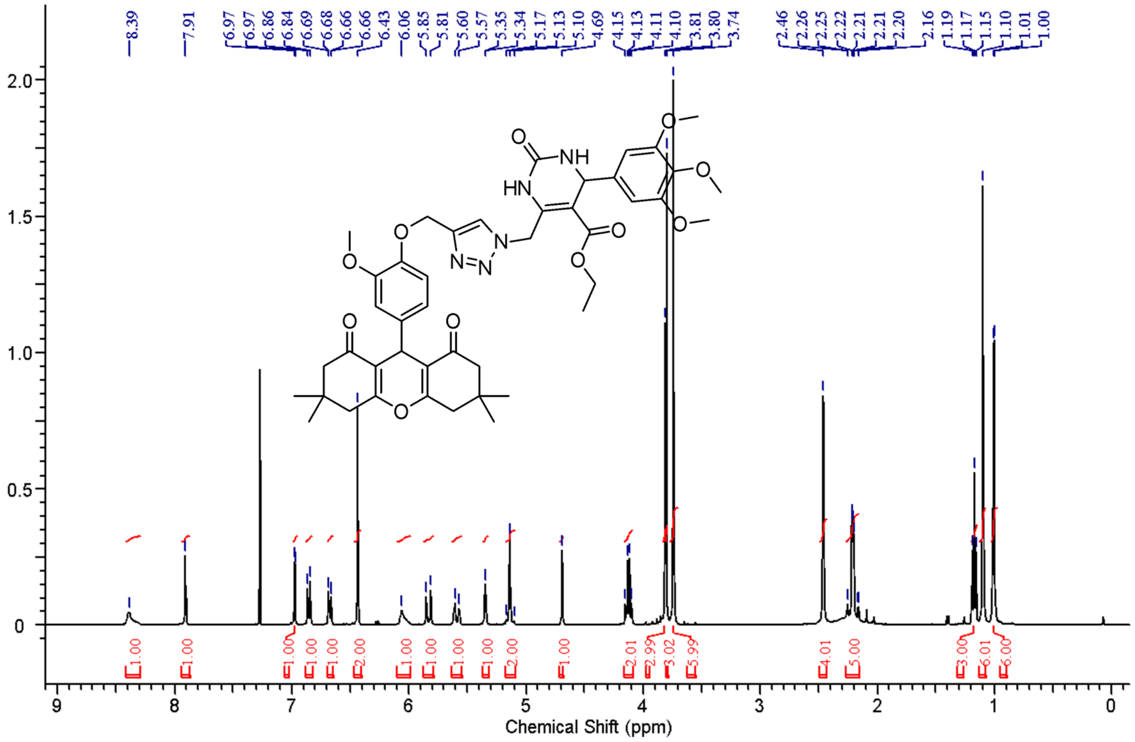

Figure S5. ^{1}H NMR (400 MHz, DMSO-*d*6) of Hybrid Compound **SJ025**

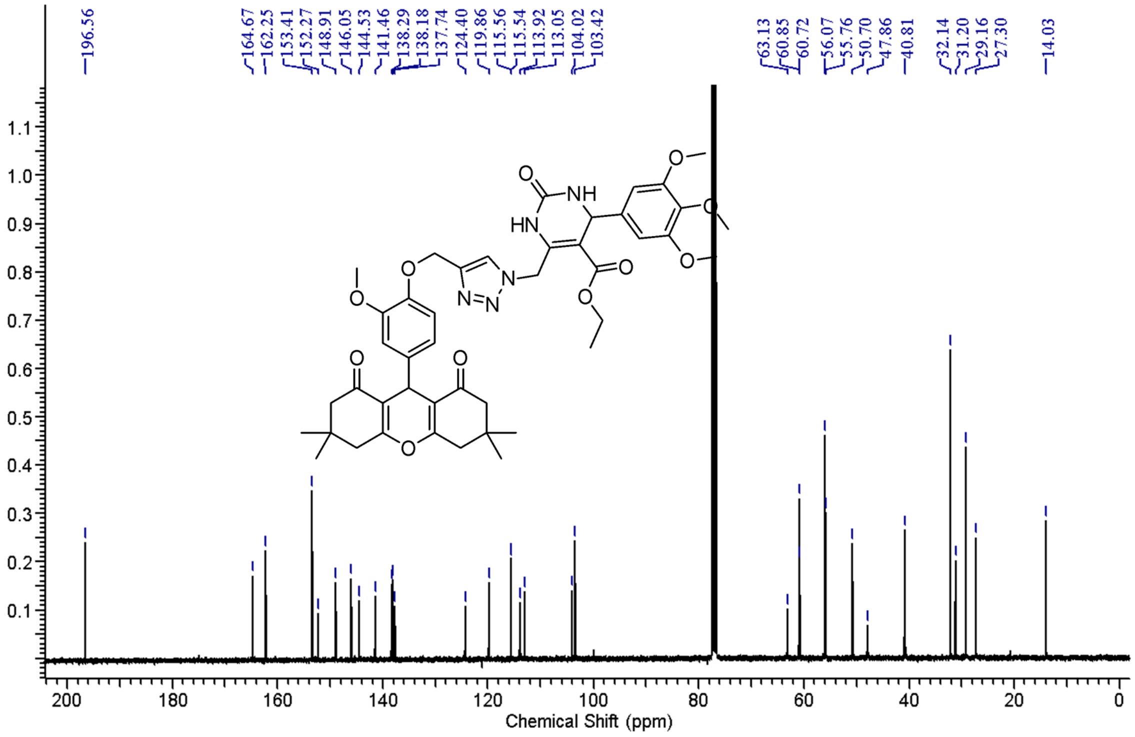

Figure S6: ^{13}C NMR (100 MHz, $CDCl_3$) of Hybrid Compound **SJ025**

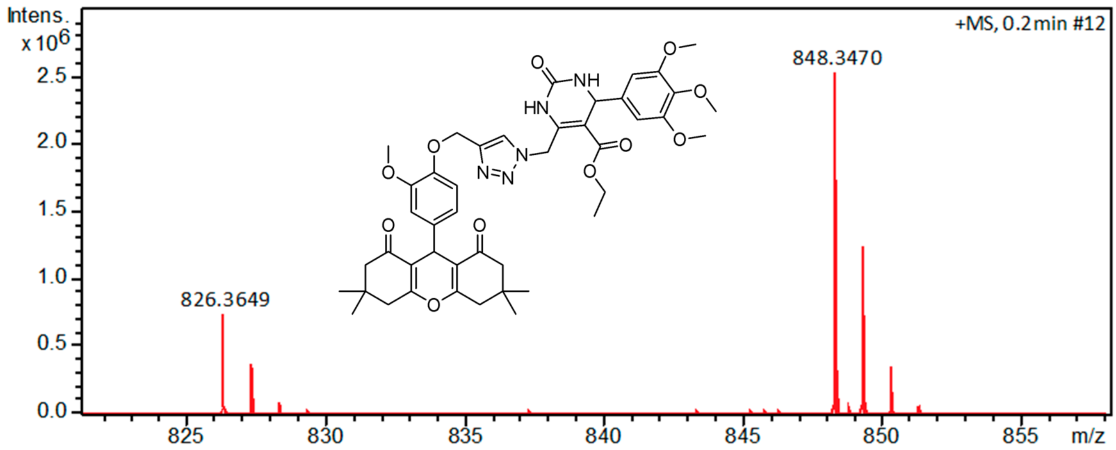

Meas. m/z	Ion Formula	m/z	err [ppm]	mSigma	rdb	e⁻ Conf	N-Rule
826.3649	$C_{44}H_{52}N_5O_{11}$	826.3658	1	7.1	21.5	even	ok
848.347	$C_{44}H_{51}N_5NaO_{11}$	848.3477	0.8	6.6	21.5	even	ok

Figure S7. HRMS of Hybrid Compound **SJ025**

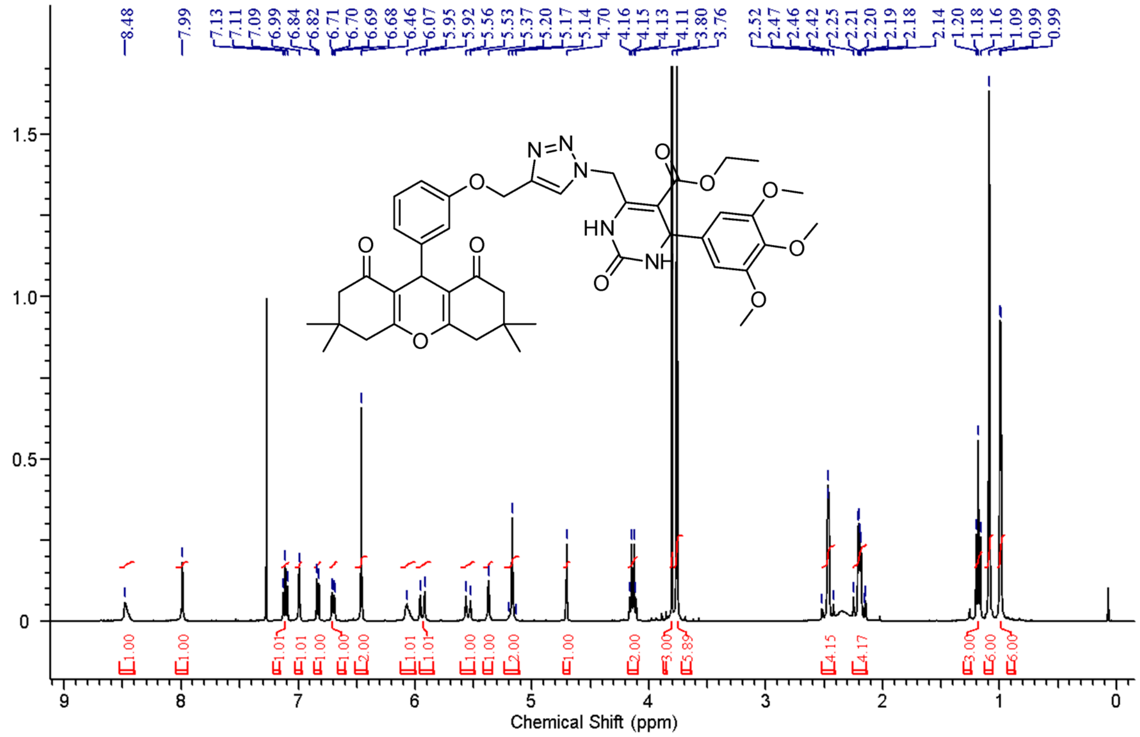

Figure S8. ^{1}H NMR (400 MHz, DMSO-*d*6) of Hybrid Compound **SJ028**

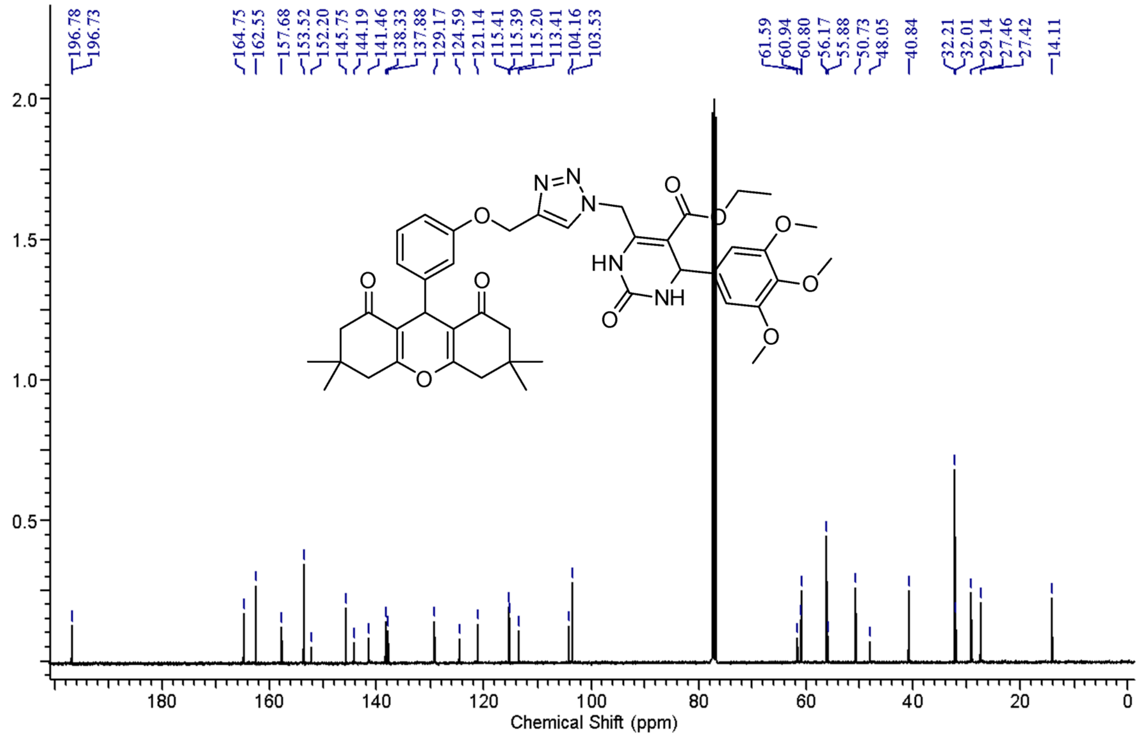

Figure S9: ^{13}C NMR (100 MHz, $CDCl_3$) of Hybrid Compound **SJ028**

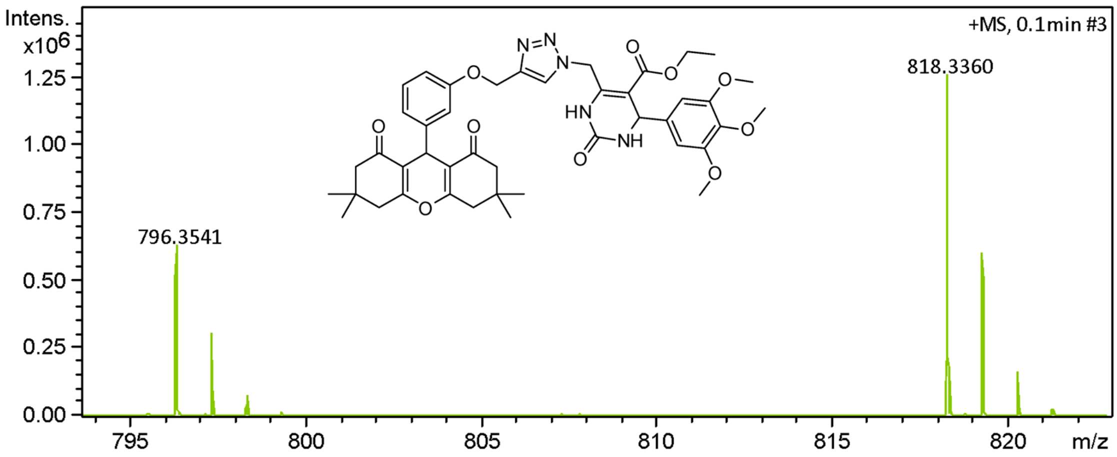

Meas. m/z	Ion Formula	m/z	err [ppm]	mSigma	rdb	e⁻ Conf	N-Rule
796.3541	$C_{43}H_{50}N_5O_{10}$	796.3552	1.4	8.3	21.5	even	ok
818.336	$C_{43}H_{49}N_5NaO_{10}$	818.3372	1.4	7.9	21.5	even	ok

Figure S10. HRMS of Hybrid Compound **SJ028**

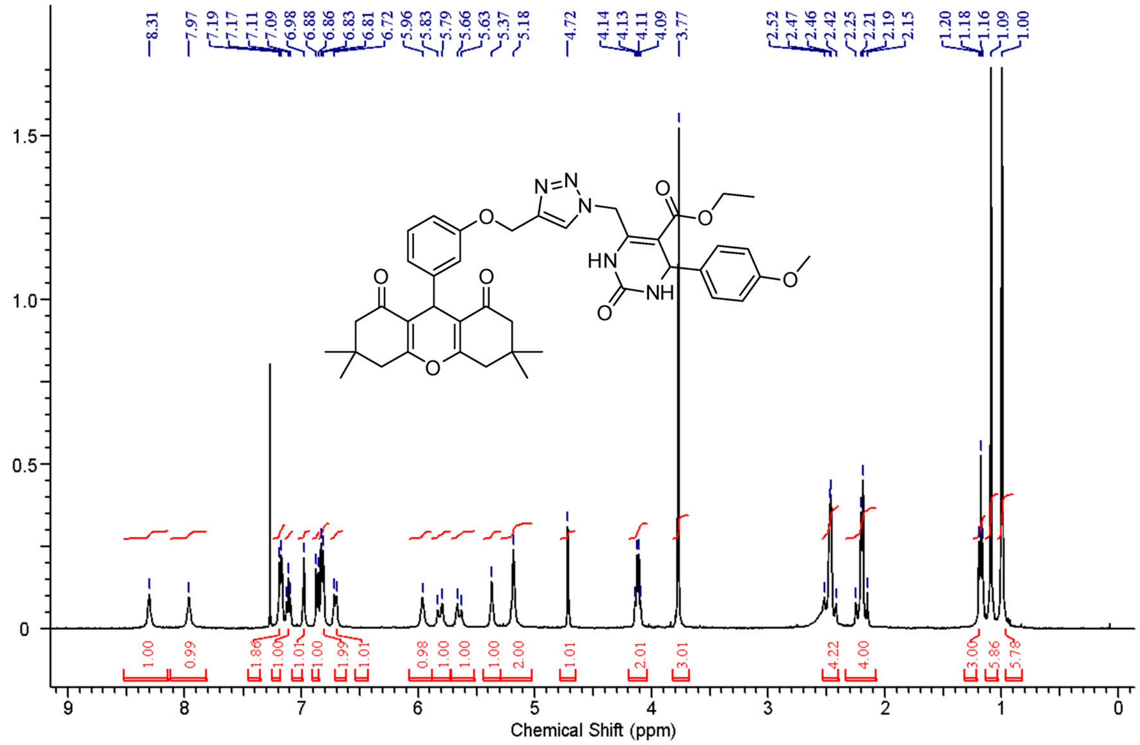

Figure S11. ^{1}H NMR (400 MHz, $CDCl_3$) of Hybrid Compound **SJ064**

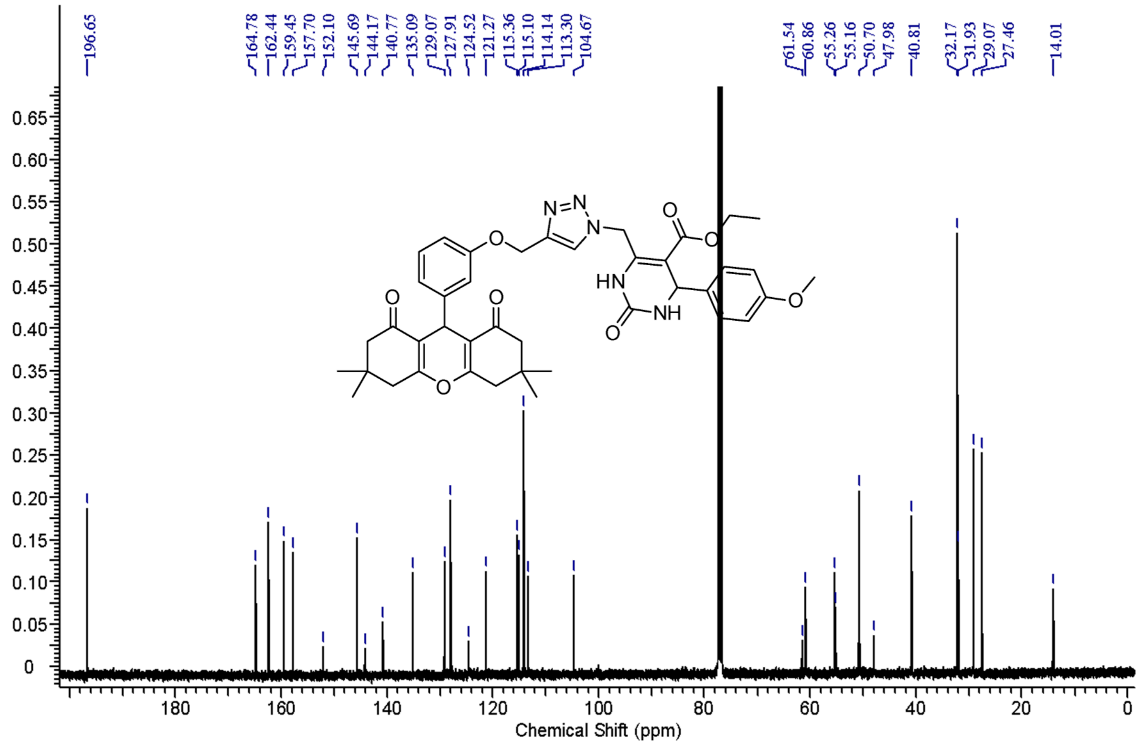

Figure S12: ^{13}C NMR (100 MHz, $CDCl_3$) of Hybrid Compound **SJ064**

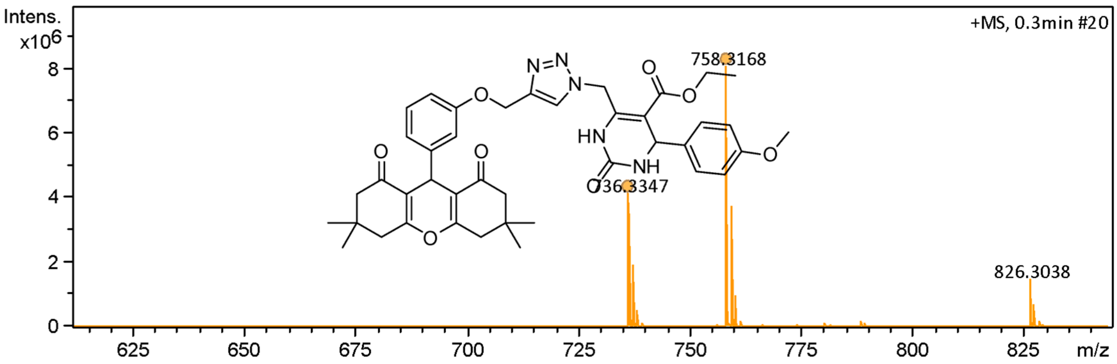

Meas. m/z	Ion Formula	m/z	err [ppm]	mSigma	rdb	e⁻ Conf	N-Rule
736.3347	$C_{41}H_{46}N_5O_8$	736.334 1	-0.8	4.5	21.5	even	ok
758.3168	$C_{41}H_{45}N_5NaO_8$	758.316 0	-1.0	6.3	21.5	even	ok

Figure S13. HRMS of Hybrid Compound **SJ064**

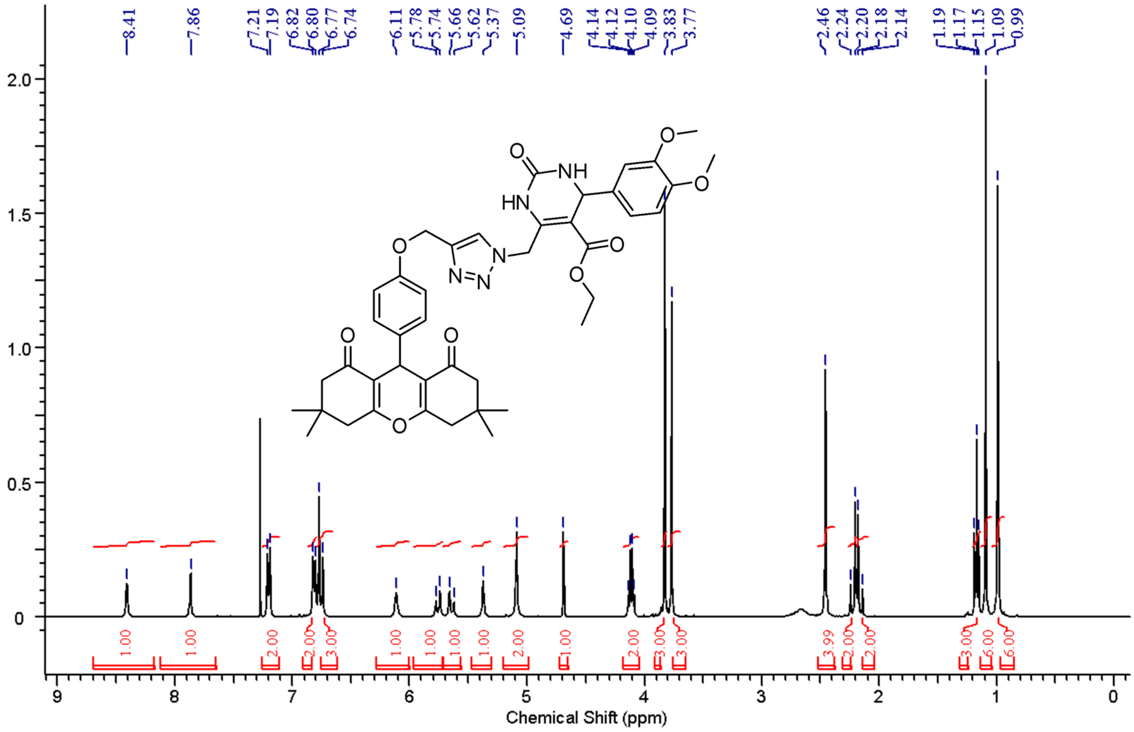

Figure S14. ^{1}H NMR (400 MHz, $CDCl_3$) of Hybrid Compound **SJ077**

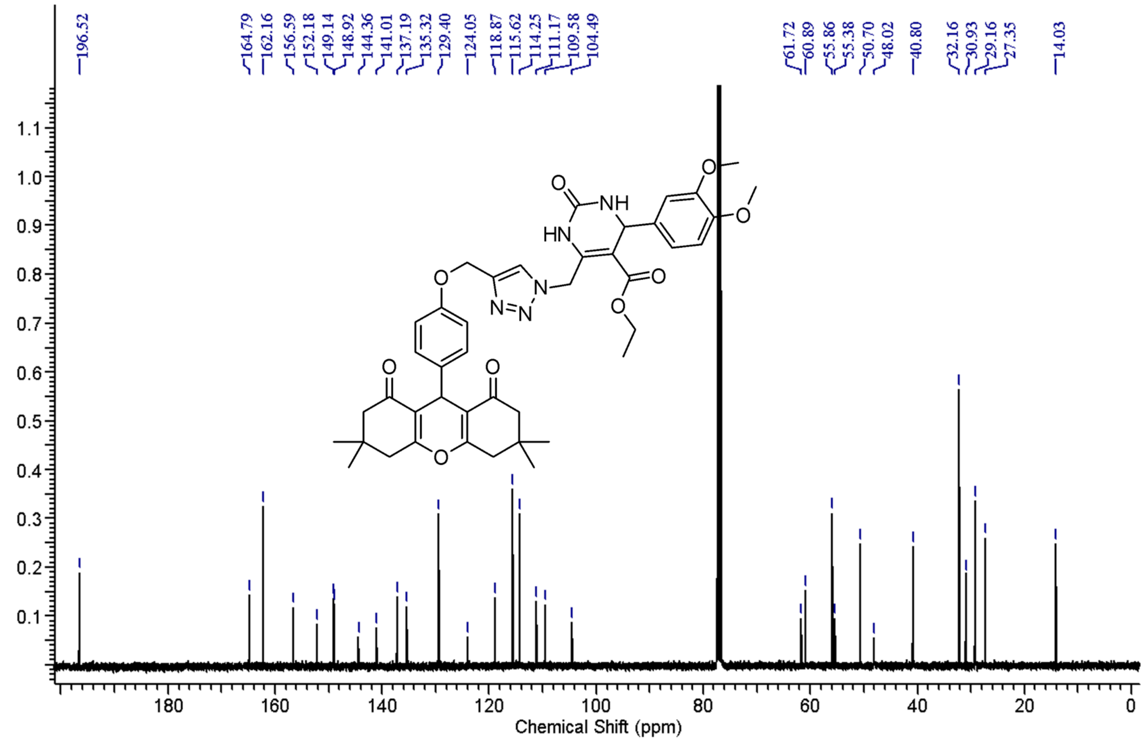

Figure S15: ^{13}C NMR (100 MHz, $CDCl_3$) of Hybrid Compound **SJ077**

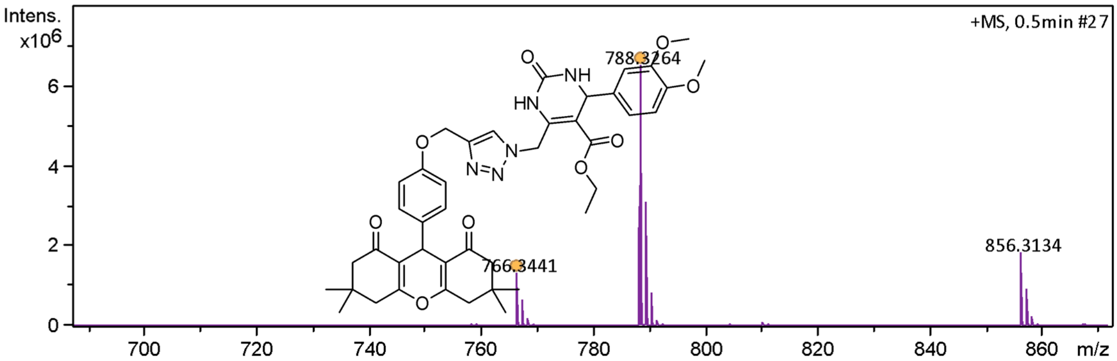

Meas. m/z	Ion Formula	m/z	err [ppm]	mSigma	rdb	e⁻ Conf	N-Rule
766.3441	$C_{42}H_{48}N_5O_9$	766.3447	0.7	3.5	21.5	even	ok
788.3264	$C_{42}H_{47}N_5NaO_9$	788.3266	0.3	4.4	21.5	even	ok

Figure S16. HRMS of Hybrid Compound **SJ077**

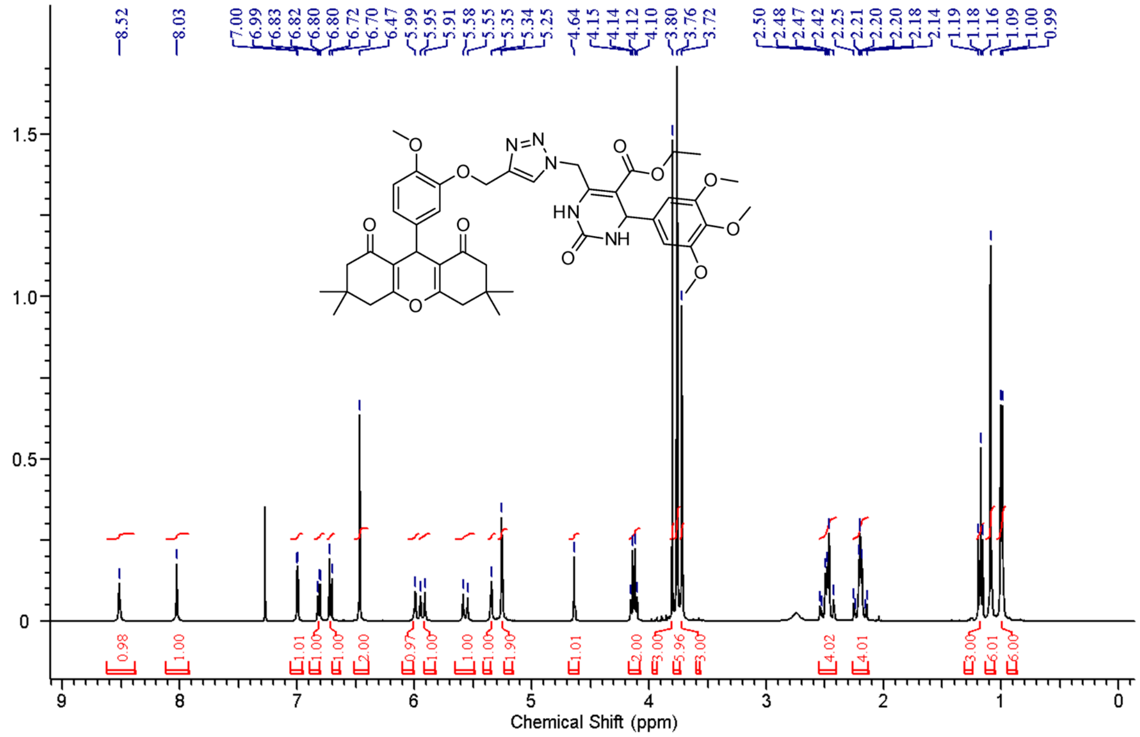

Figure S17: ^{13}C NMR (100 MHz, CDCl$_3$) of Hybrid Compound **SJ078**

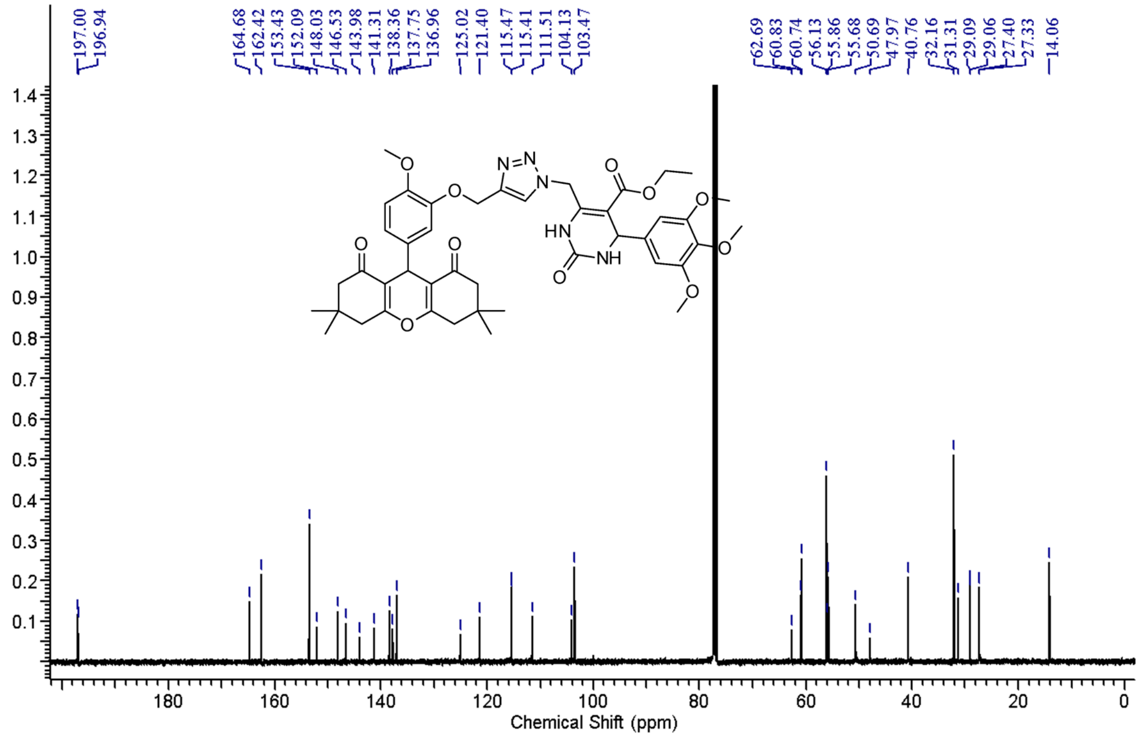

Figure S18: ^{13}C NMR (100 MHz, CDCl$_3$) of Hybrid Compound **SJ078**

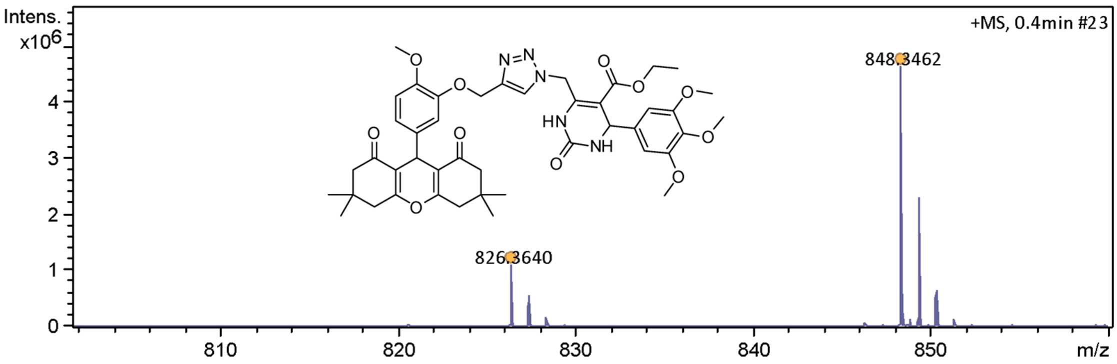

Meas. m/z	Ion Formula	m/z	err [ppm]	mSigma	rdb	e⁻ Conf	N-Rule
826.3640	$C_{44}H_{52}N_5O_{11}$	826.3658	2.1	2.1	21.5	even	ok
848.3462	$C_{44}H_{51}N_5NaO_{11}$	848.3477	1.8	5.7	21.5	even	ok

Figure S19. HRMS of Hybrid Compound **SJ078**

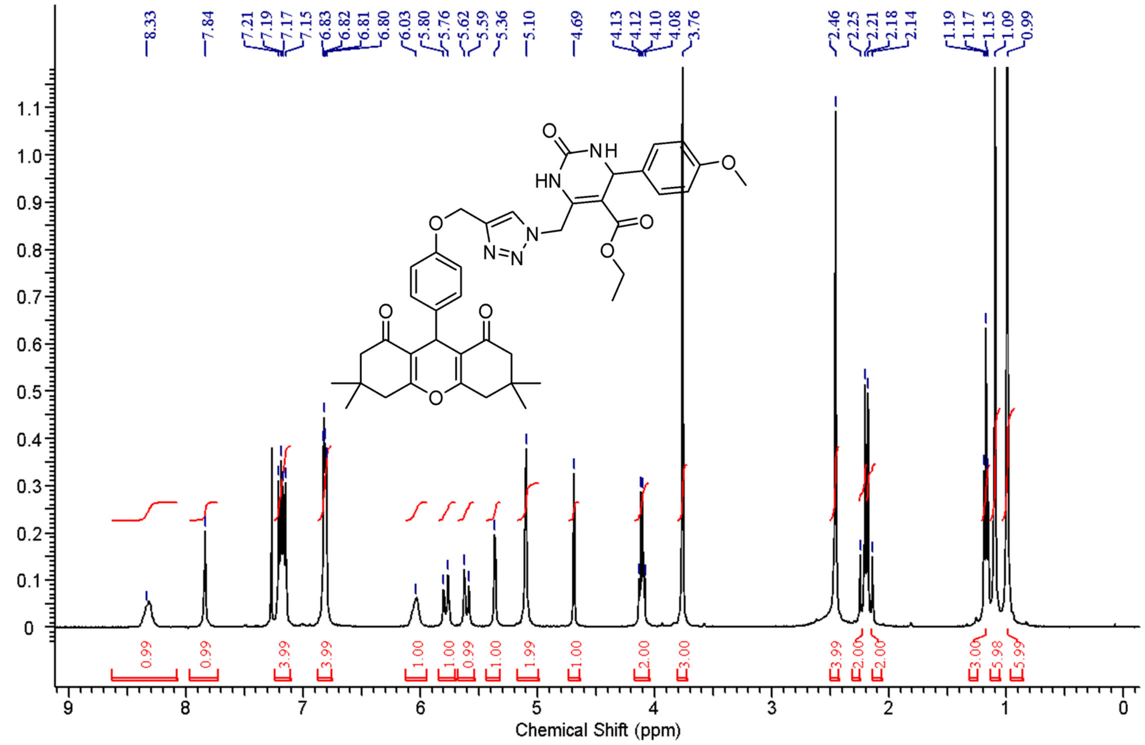

Figure S20: ^{1}H NMR (400 MHz, $CDCl_3$) of Hybrid Compound **SJ079**

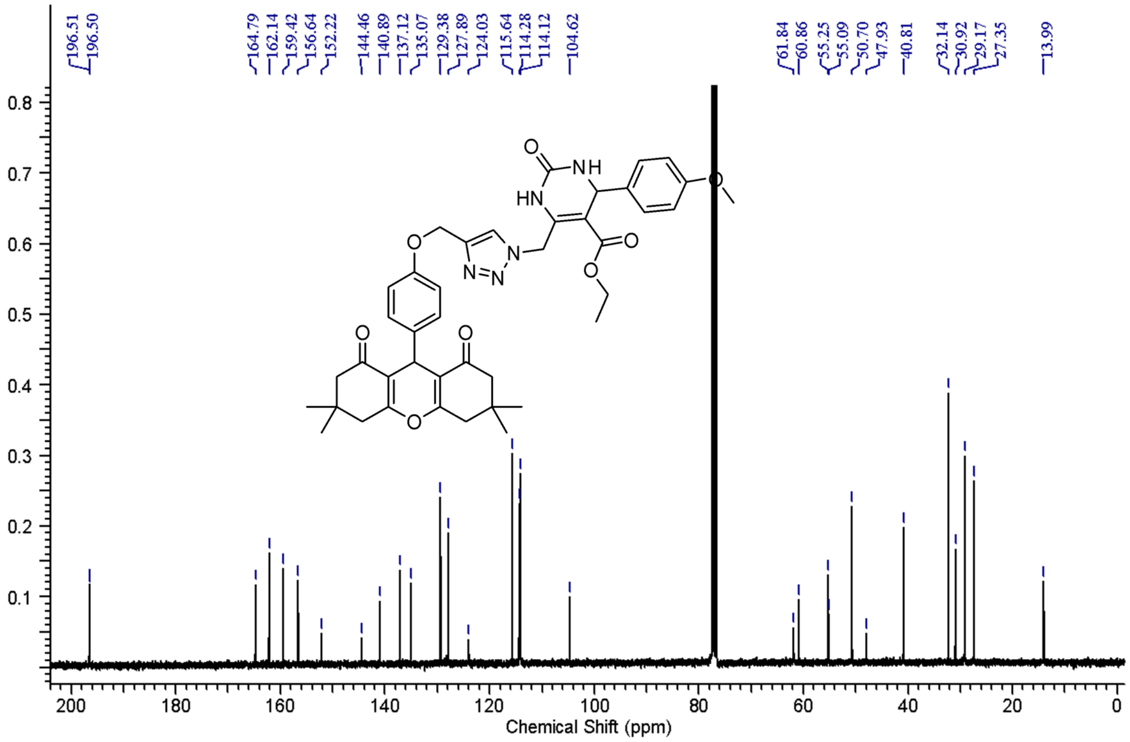

Figure S21: ^{13}C NMR (100 MHz, $CDCl_3$) of Hybrid Compound **SJ079**

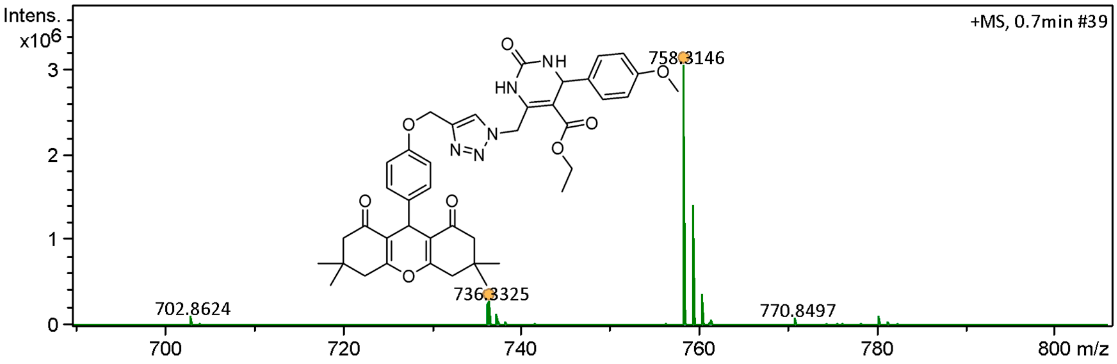

Meas. m/z	Ion Formula	m/z	err [ppm]	mSigma	rdb	e⁻ Conf	N-Rule
736.3325	$C_{41}H_{46}N_5O_8$	736.3341	2.1	4.1	21.5	even	ok
758.3146	$C_{41}H_{45}N_5NaO_8$	758.3160	1.9	6.2	21.5	even	ok

Figure S22. HRMS of Hybrid Compound **SJ079**

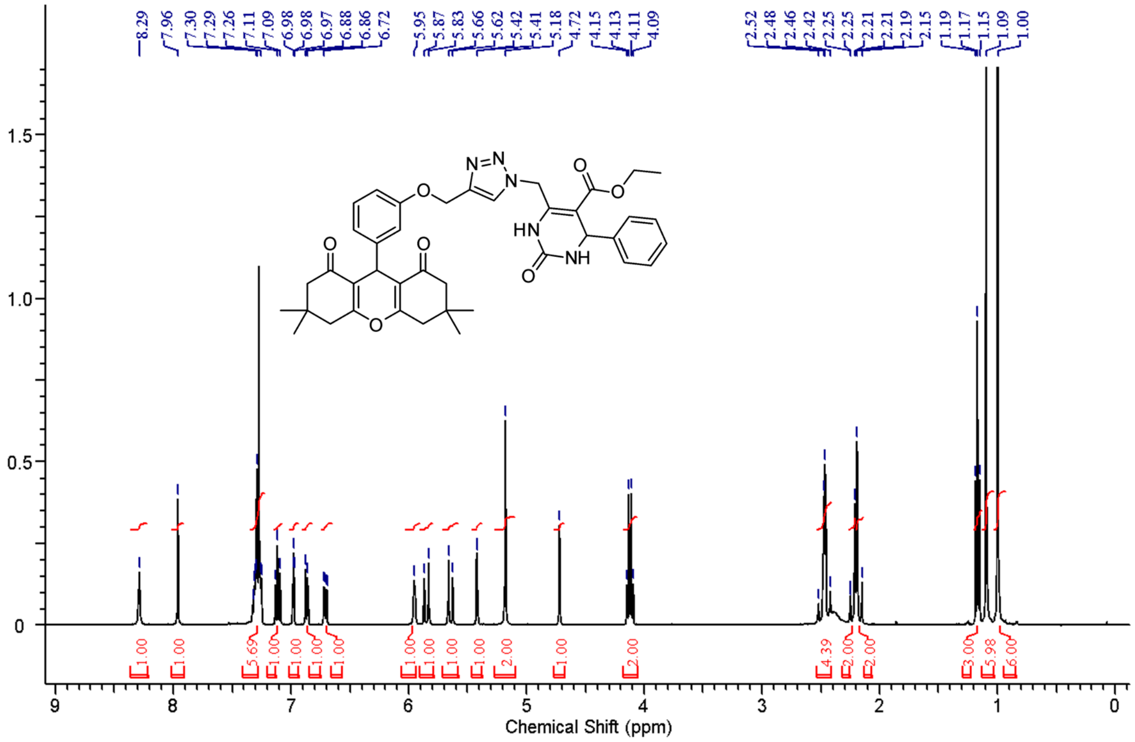

Figure S23: ^{1}H NMR (400 MHz, $CDCl_3$) of Hybrid Compound **SJ080**

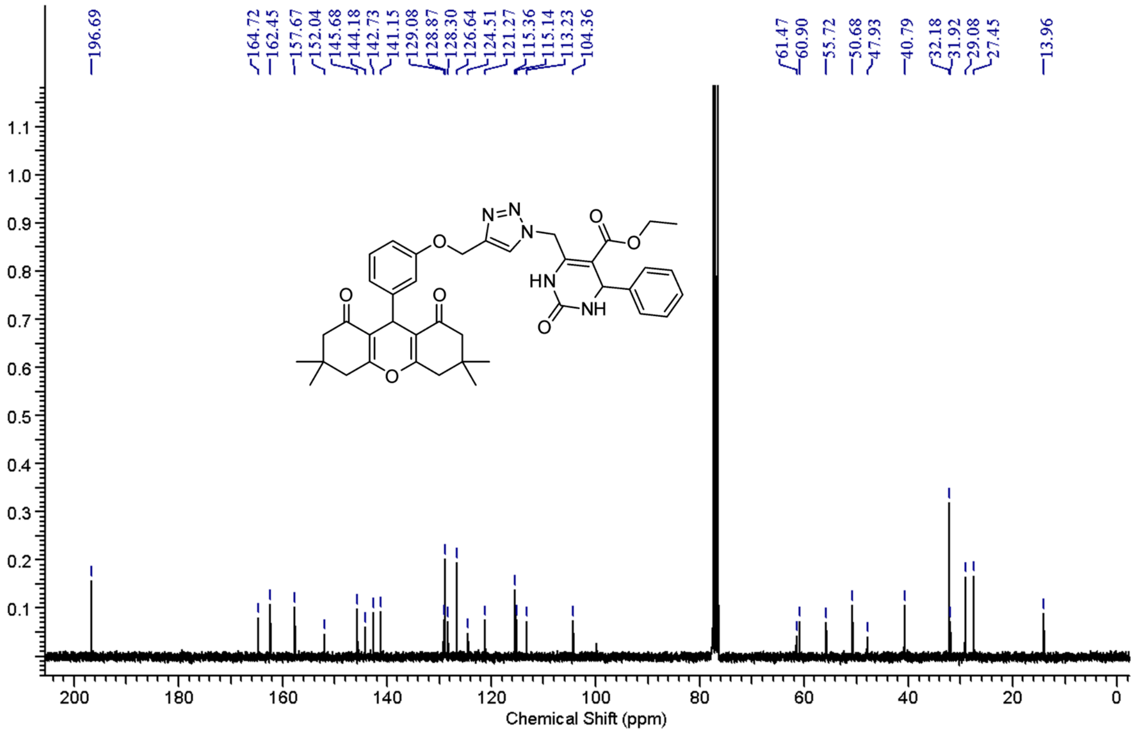

Figure S24: ^{13}C NMR (100 MHz, $CDCl_3$) of Hybrid Compound **SJ080**

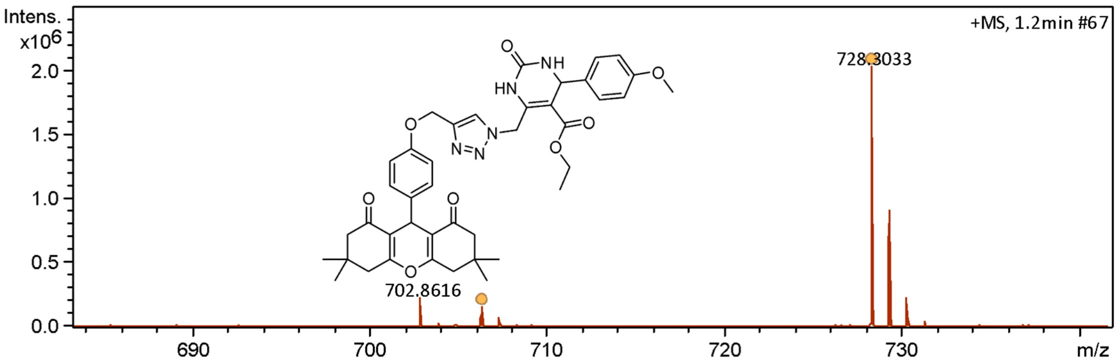

Meas. m/z	Ion Formula	m/z	err [ppm]	mSigma	rdb	e⁻ Conf	N-Rule
706.3213	$C_{40}H_{44}N_5O_7$	706.3235	3.2	14.8	21.5	even	ok
728.3033	$C_{40}H_{43}N_5NaO_7$	728.3055	3.0	5.6	21.5	even	ok

Figure S25. HRMS of Hybrid Compound **SJ080**

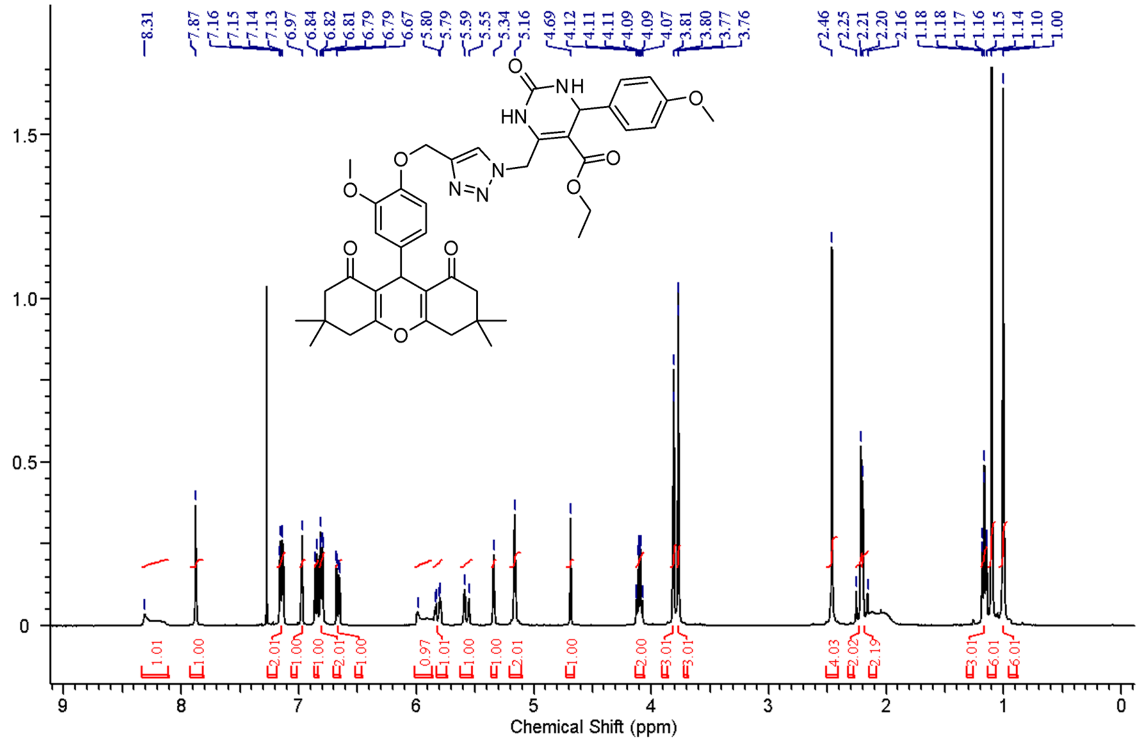

Figure S26: ^{1}H NMR (400 MHz, $CDCl_3$) of Hybrid Compound **SJ099**

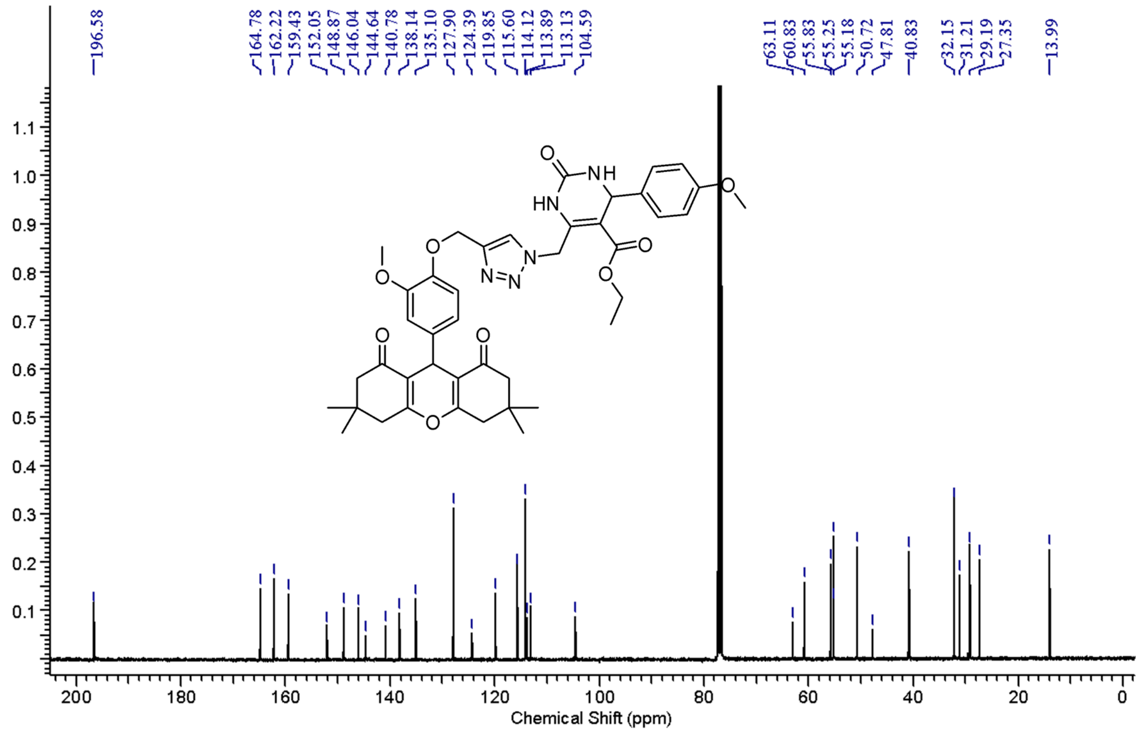

Figure S27: ^{13}C NMR (100 MHz, $CDCl_3$) of Hybrid Compound **SJ099**

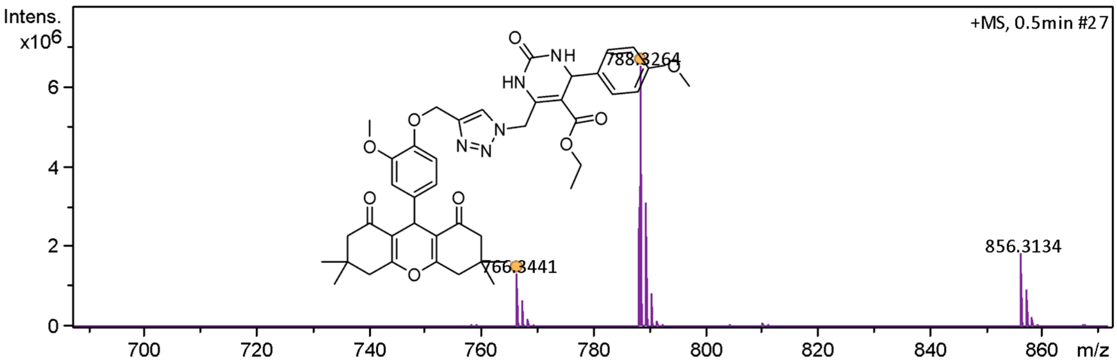

Meas. m/z	Ion Formula	m/z	err [ppm]	mSigma	rdb	e⁻ Conf	N-Rule
766.3441	$C_{42}H_{48}N_5O_9$	766.3447	0.7	3.5	21.5	even	ok
788.3264	$C_{42}H_{47}N_5NaO_9$	788.3266	0.3	4.4	21.5	even	ok

Figure S28. HRMS of Hybrid Compound **SJ099**

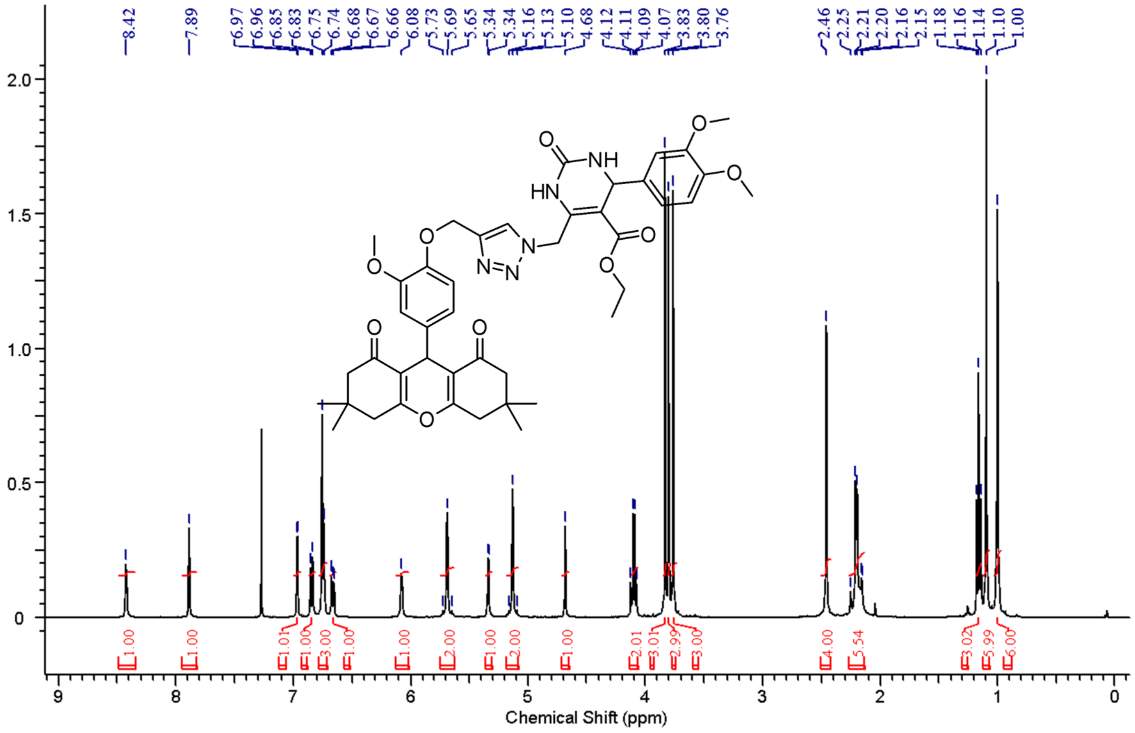

Figure S29: ^{1}H NMR (400 MHz, $CDCl_3$) of Hybrid Compound **SJ100**

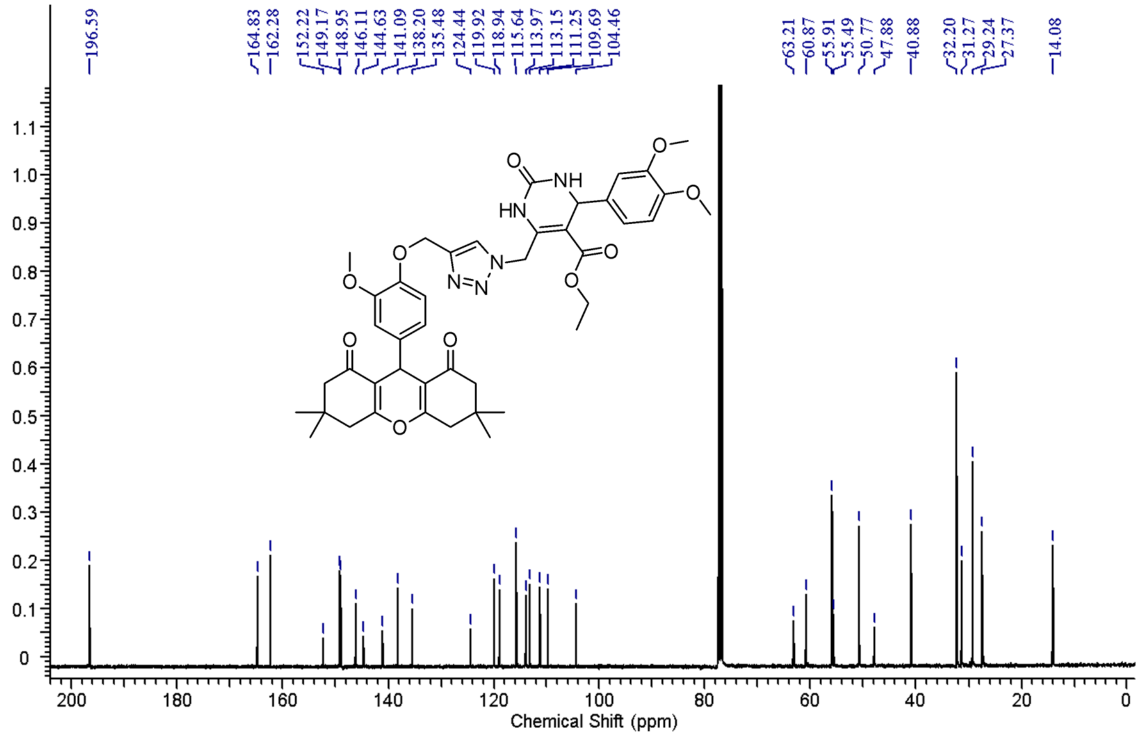

Figure S30: ^{13}C NMR (100 MHz, $CDCl_3$) of Hybrid Compound **SJ100**

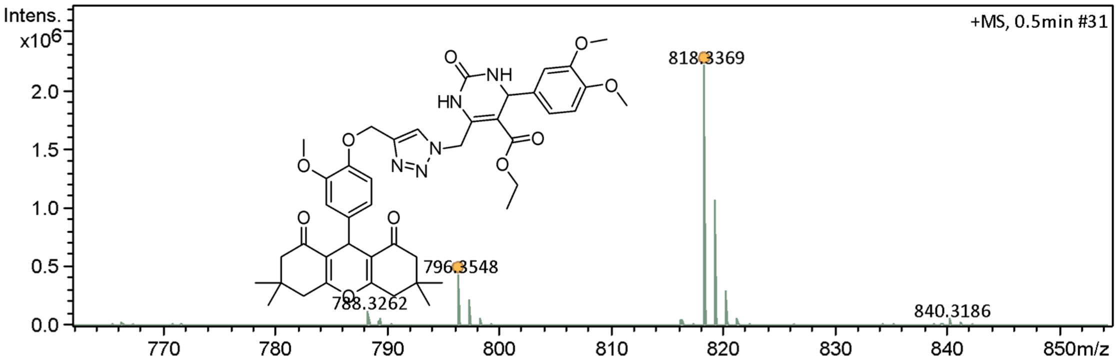

Meas. m/z	Ion Formula	m/z	err [ppm]	mSigma	rdb	e¯ Conf	N-Rule
796.3548	$C_{43}H_{50}N_5O_{10}$	796.3552	0.5	5.2	21.5	even	ok
818.3369	$C_{43}H_{49}N_5NaO_{10}$	818.3372	0.3	6.8	21.5	even	ok

Figure S31. HRMS of Hybrid Compound **SJ100**

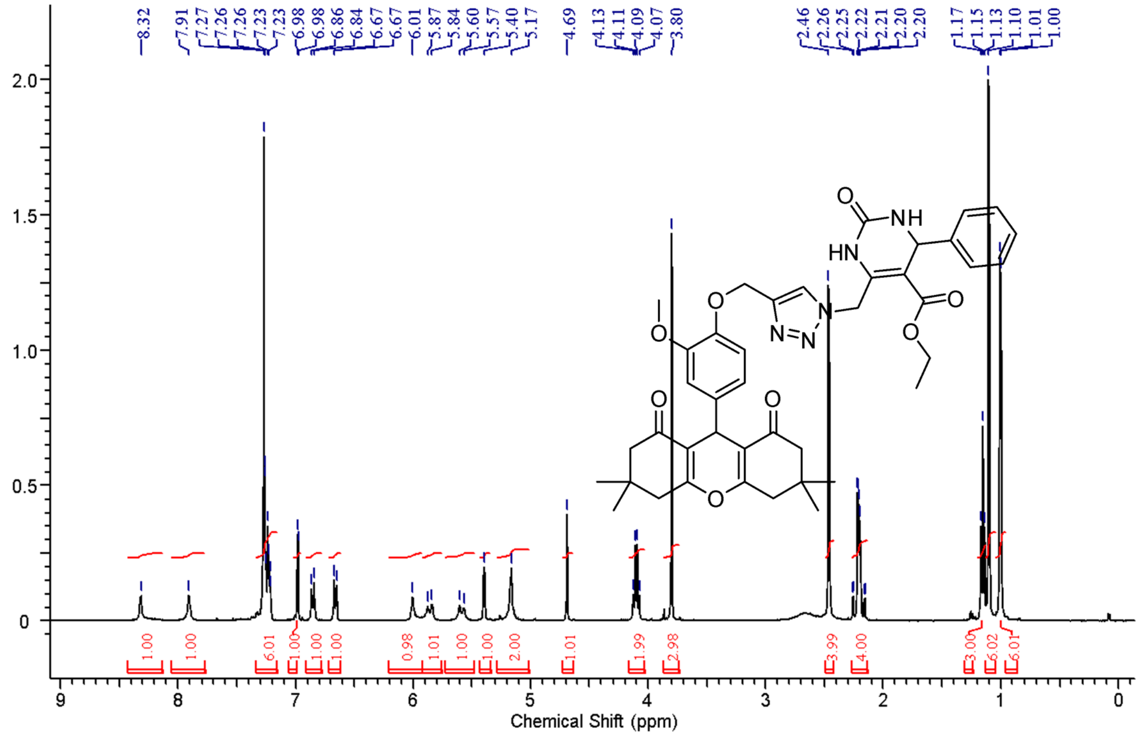

Figure S32: ^{1}H NMR (400 MHz, $CDCl_3$) of Hybrid Compound **SJ103**

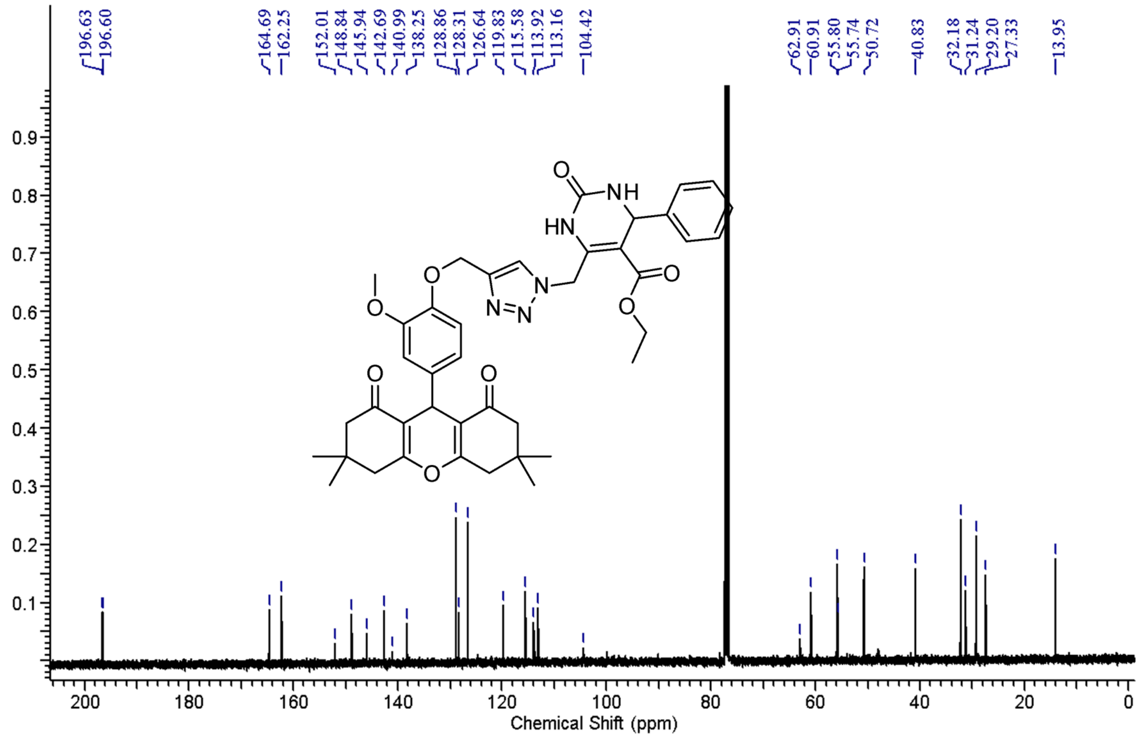

Figure S33: ^{13}C NMR (100 MHz, $CDCl_3$) of Hybrid Compound **SJ103**

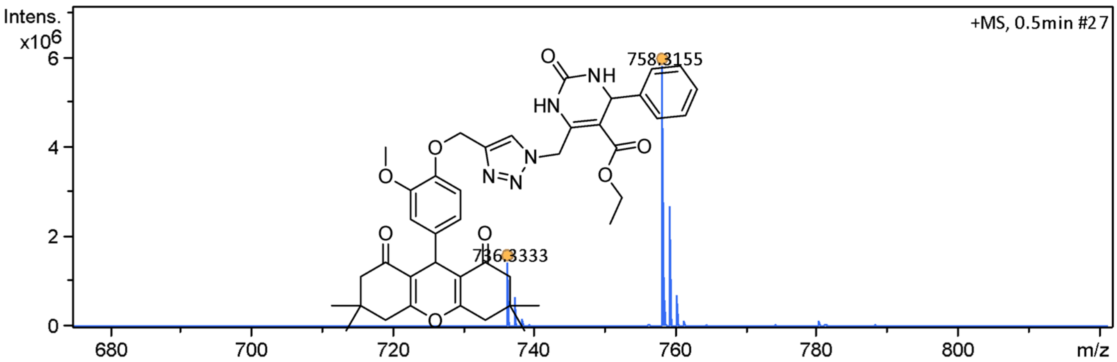

Meas. m/z	Ion Formula	m/z	err [ppm]	mSigma	rdb	e¯ Conf	N-Rule
758.3155	C41H45N5NaO8	758.3160	0.7	4.6	21.5	even	ok
736.3333	C41H46N5O8	736.3341	1.1	11.0	21.5	even	ok

Figure S34. HRMS of Hybrid Compound **SJ103**

^{1}H NMR, ^{13}C NMR & HRMS of Pyran-Dihydropyrimidinone Hybrids

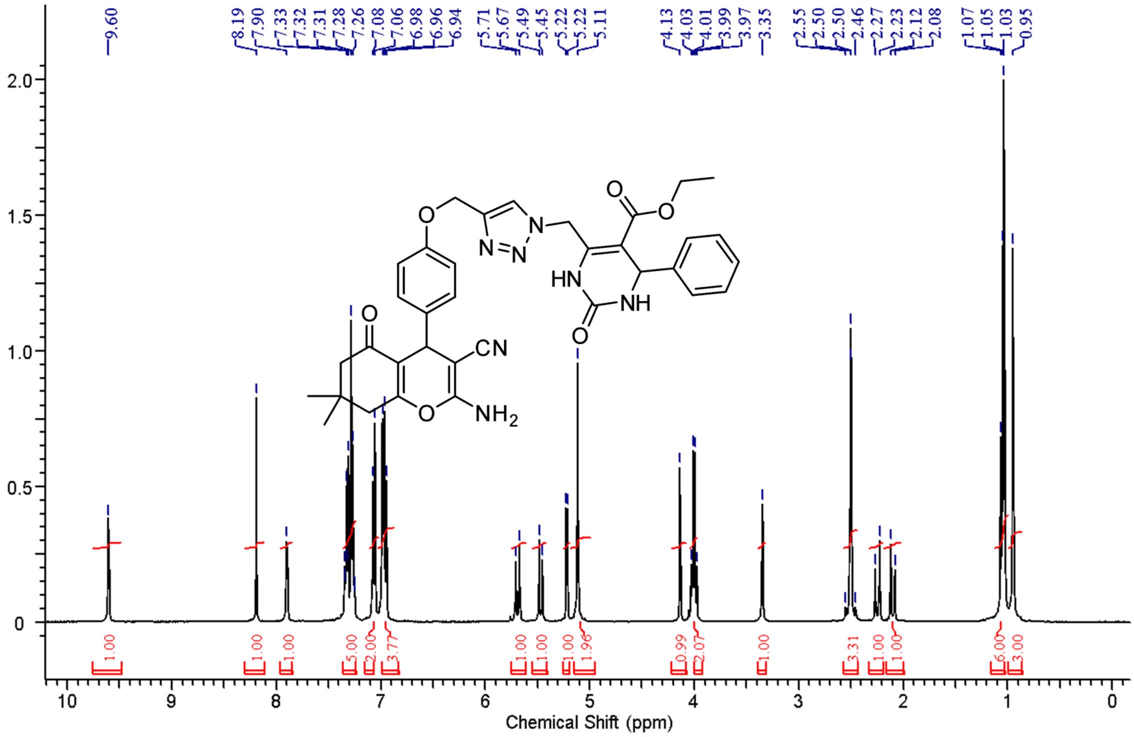

Figure S35: ^{1}H-NMR (400 MHz, DMSO-*d*6) of Hybrid Compound **SJ071**

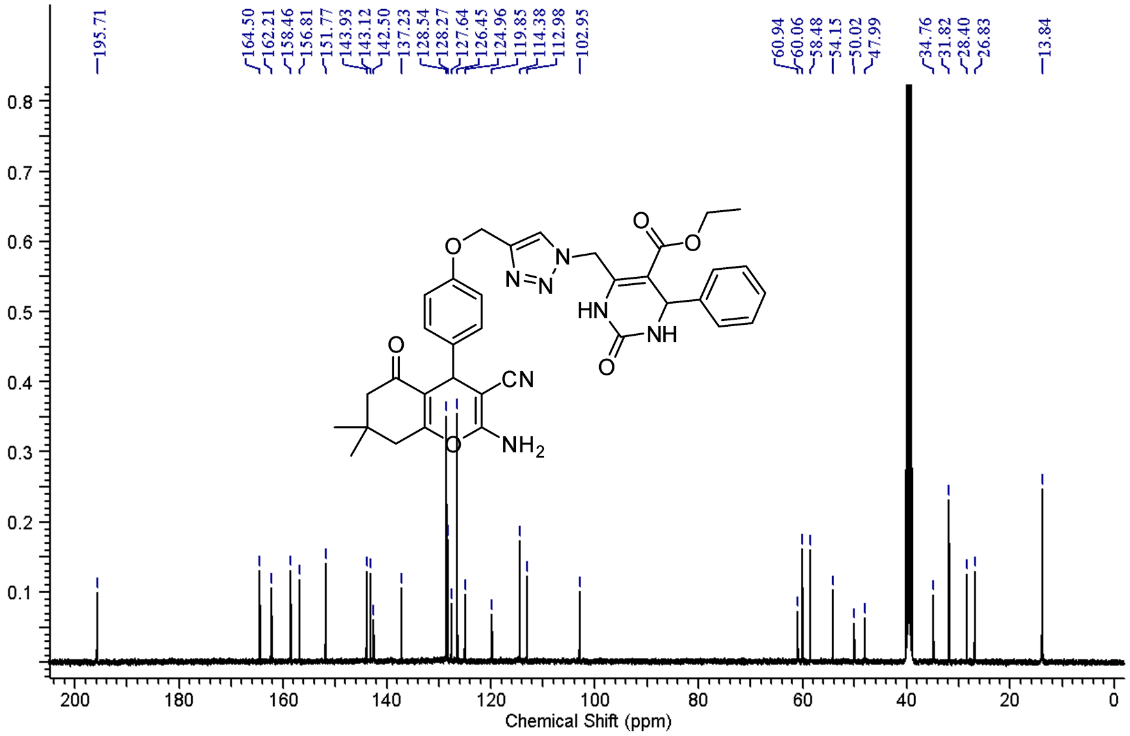

Figure S36: ^{13}C-NMR (100 MHz, DMSO-*d*6) of Hybrid Compound **SJ071**

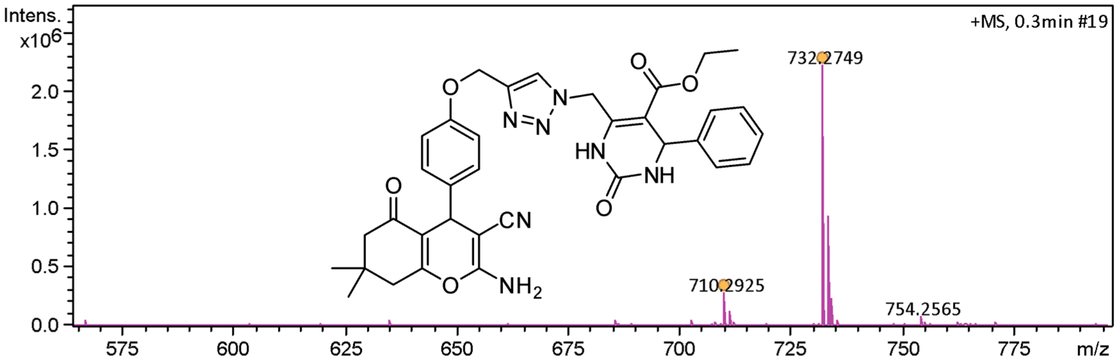

Meas. m/z	Ion Formula	m/z	err [ppm]	mSigma	rdb	e¯ Conf	N-Rule
732.2749	$C_{37}H_{39}N_7NaO_8$	732.2752	0.5	6.9	21.5	even	ok
710.2925	$C_{37}H_{40}N_7O_8$	710.2933	1.1	10.0	21.5	even	ok

Figure S37: HRMS of Hybrid Compound **SJ071**

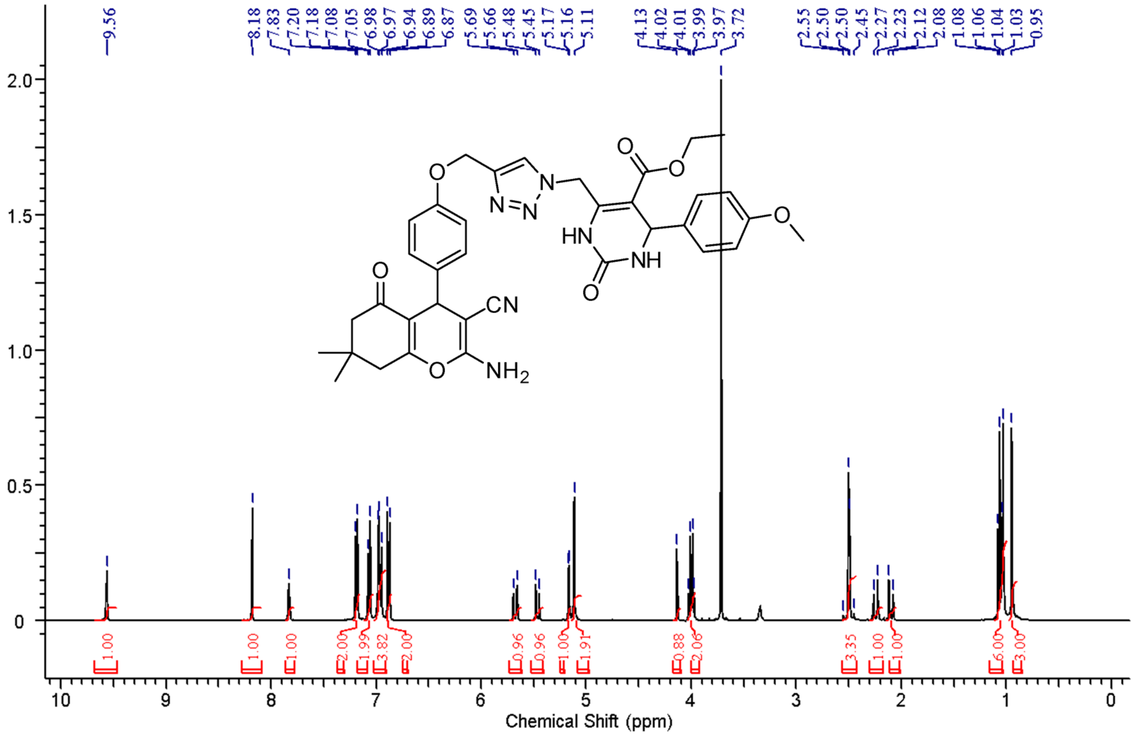

Figure S38: ^{1}H-NMR (400 MHz, DMSO-*d*6) of Hybrid Compound **SJ072**

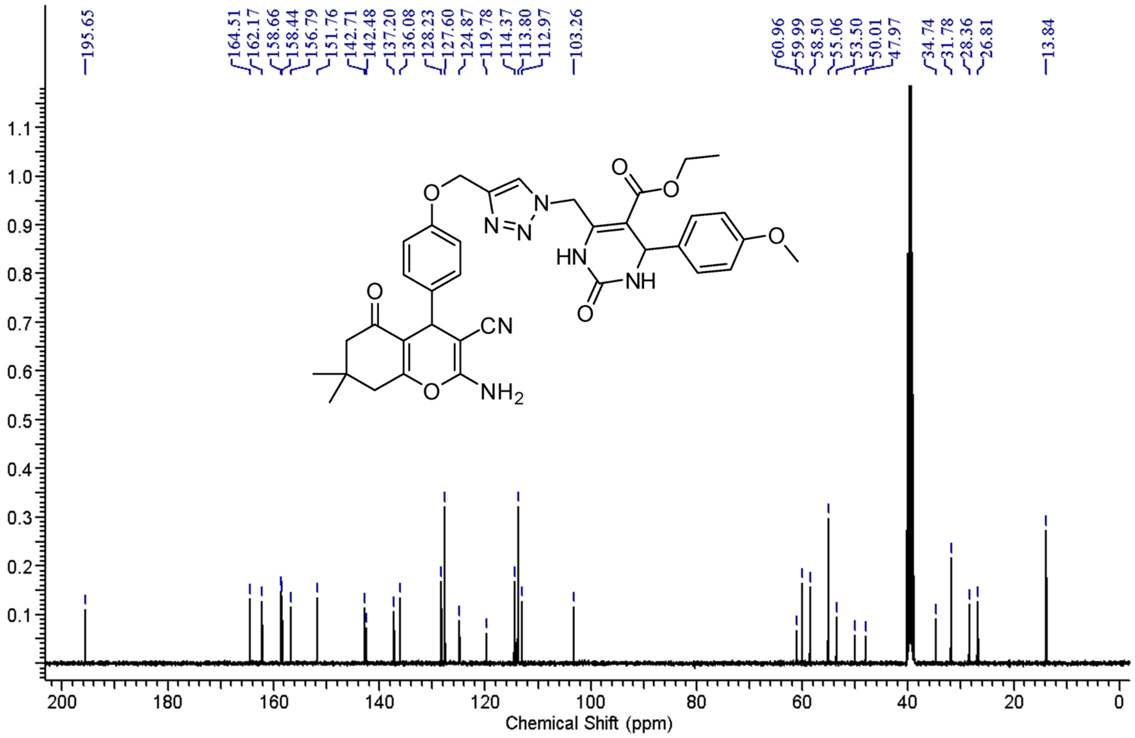

Figure S39: ^{13}C-NMR (100 MHz, DMSO-*d*6) of Hybrid Compound **SJ072**

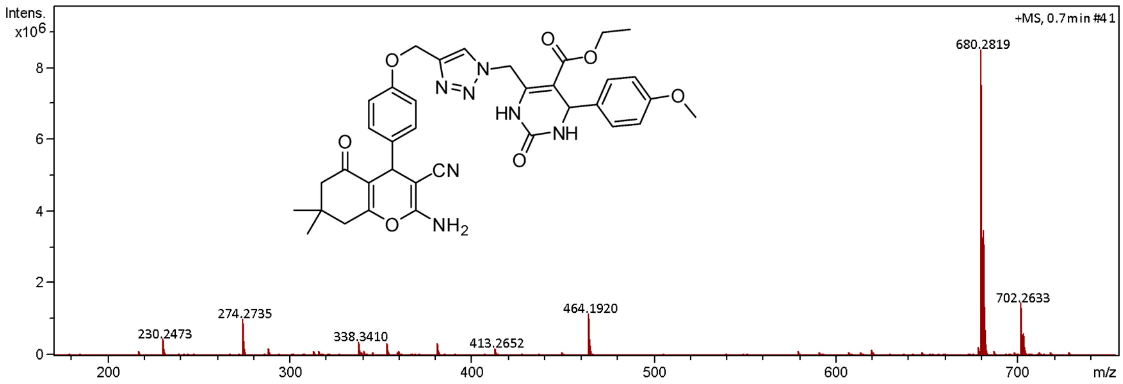

Meas. m/z	Ion Formula	m/z	err [ppm]	mSigma	rdb	e⁻ Conf	N-Rule
680.2819	$C_{36}H_{38}N_7O_7$	680.2827	1.2	7.4	21.5	even	ok
702.2633	$C_{36}H_{37}N_7NaO_7$	702.2647	2.0	7.2	21.5	even	ok

Figure S40: HRMS of Hybrid Compound **SJ072**

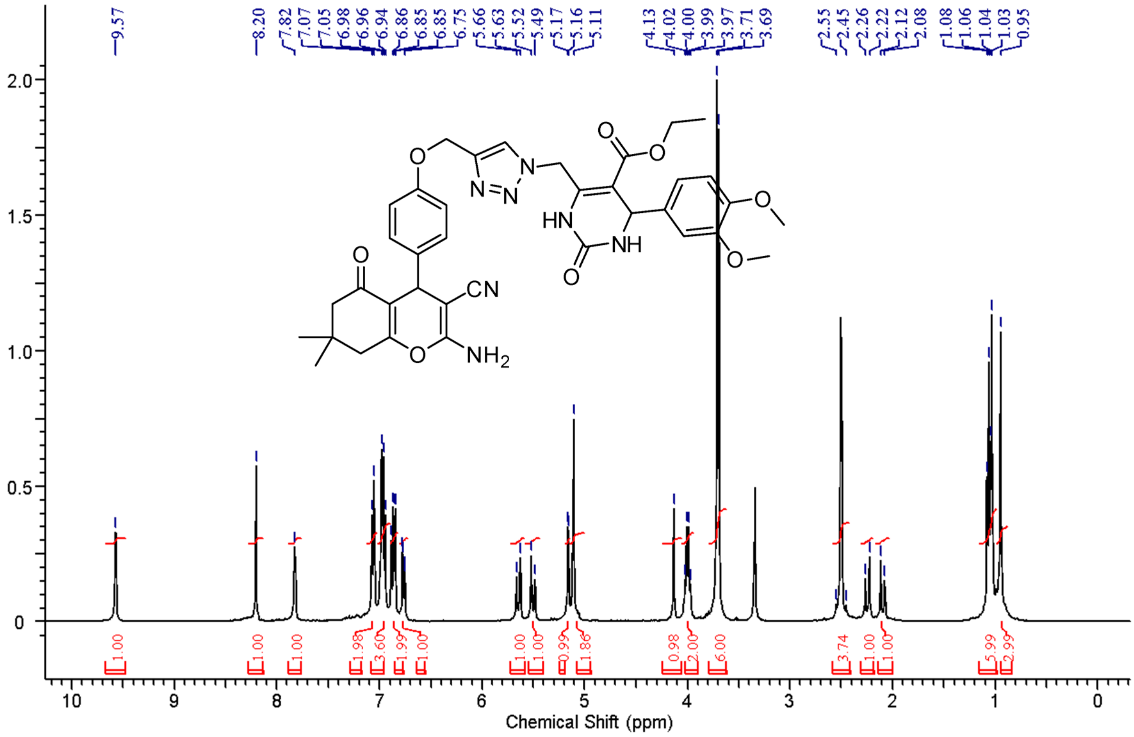

Figure S41: ^{1}H-NMR (400 MHz, DMSO-*d*6) of Hybrid Compound **SJ074**

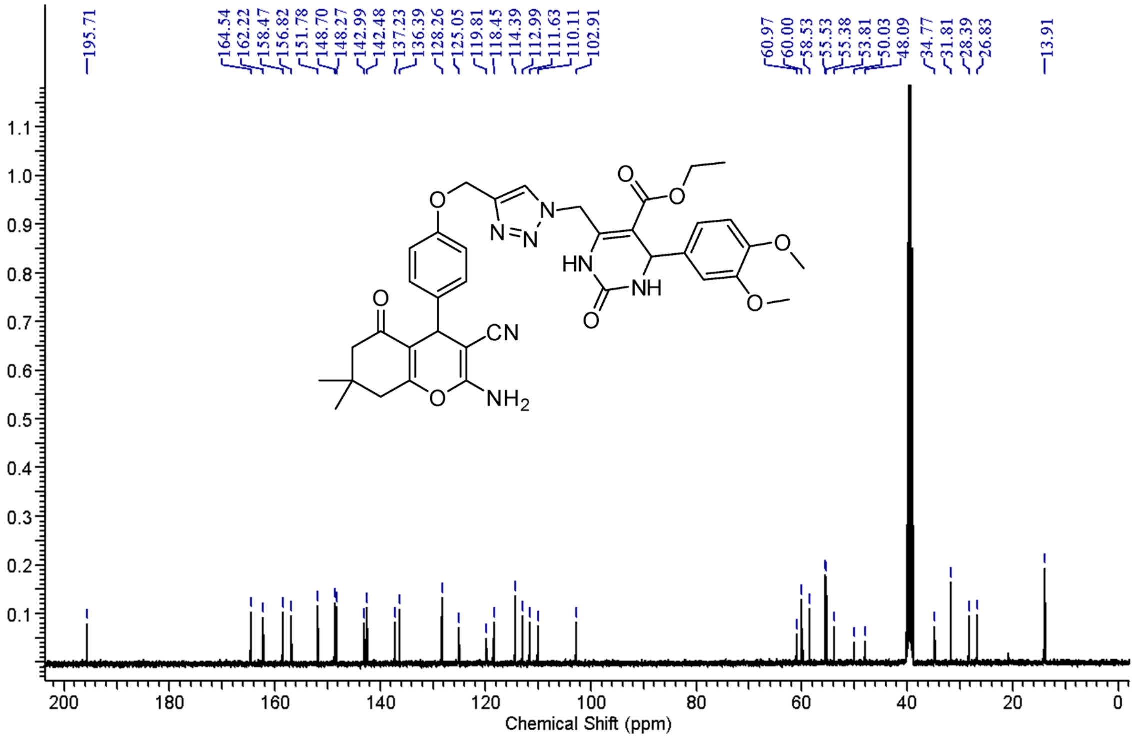

Figure S42: ^{13}C-NMR (100 MHz, DMSO-*d*6) of Hybrid Compound **SJ074**

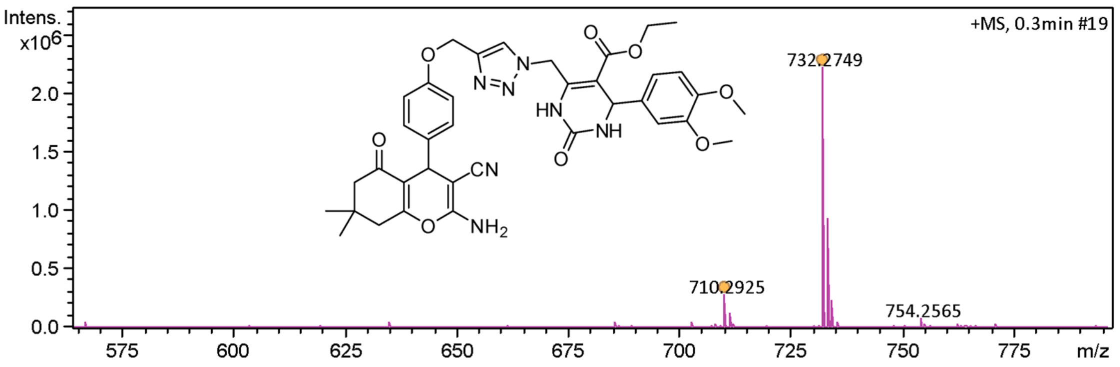

Meas. m/z	Ion Formula	m/z	err [ppm]	mSigma	rdb	e⁻ Conf	N-Rule
732.2749	$C_{37}H_{39}N7NaO_8$	732.2752	0.5	6.9	21.5	even	ok
710.2925	$C_{37}H_{40}N7O_8$	710.2933	1.1	10.0	21.5	even	ok

Figure S43: HRMS of Hybrid Compound **SJ074**

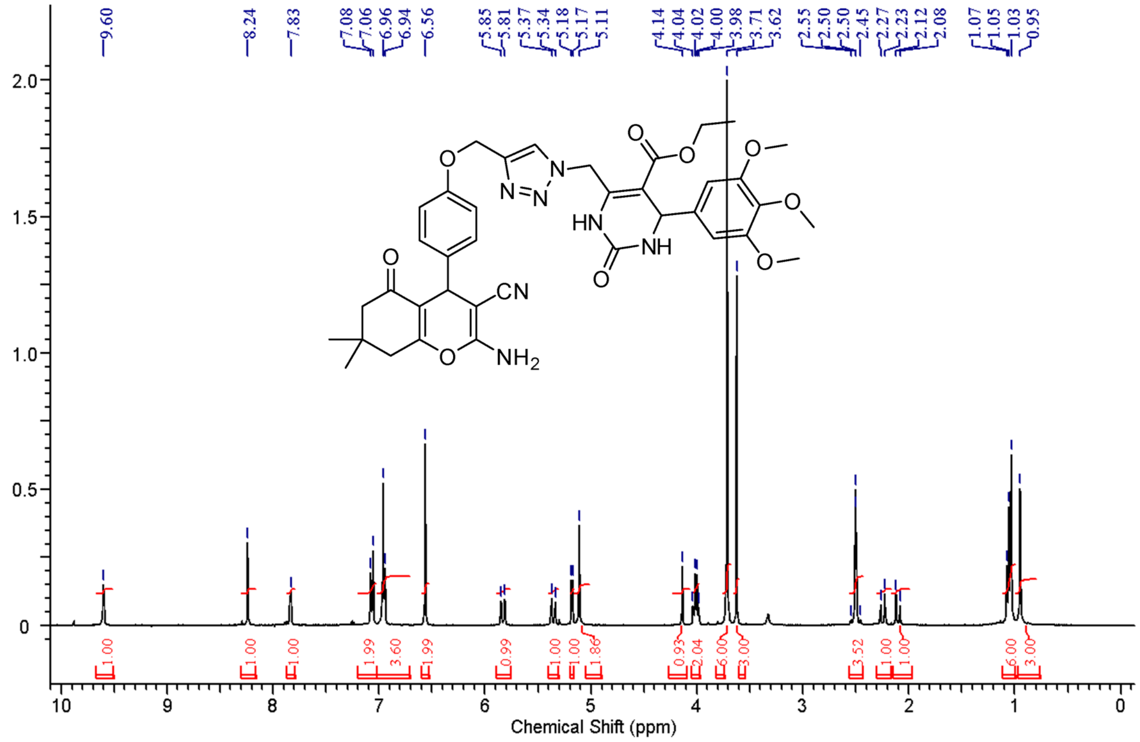

Figure S44: ^{1}H-NMR (400 MHz, DMSO-*d*6) of Hybrid Compound **SJ075**

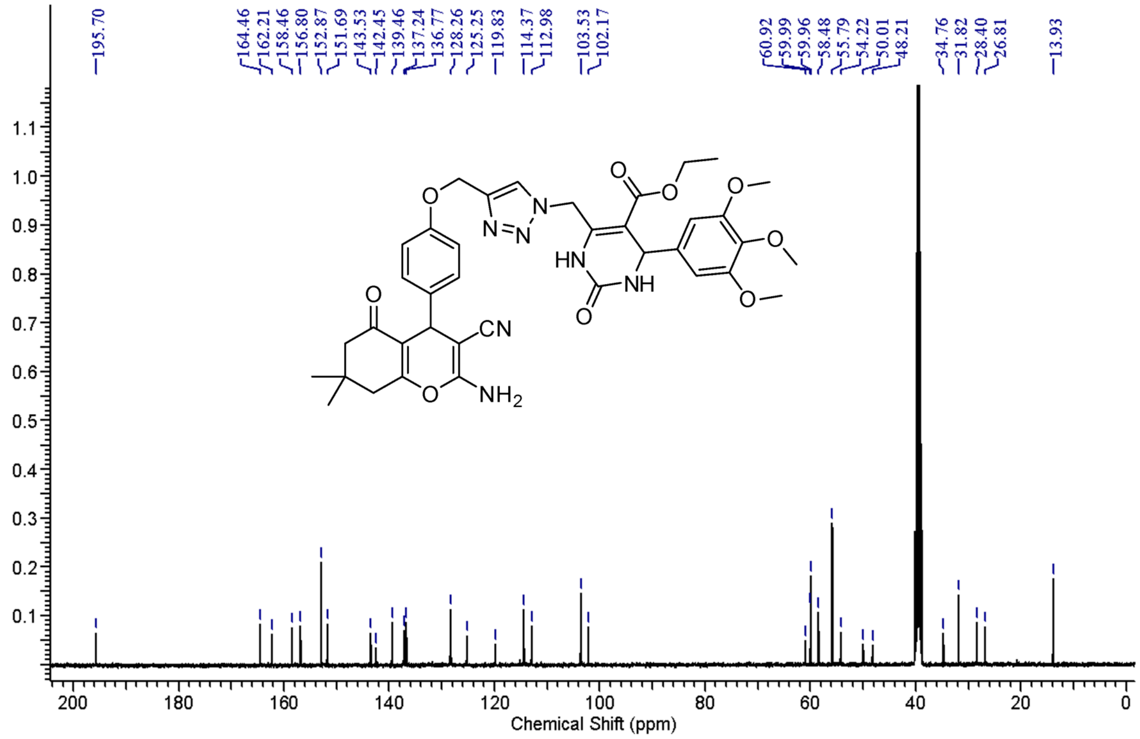

Figure S45: ^{13}C-NMR (100 MHz, DMSO-*d*6) of Hybrid Compound **SJ075**

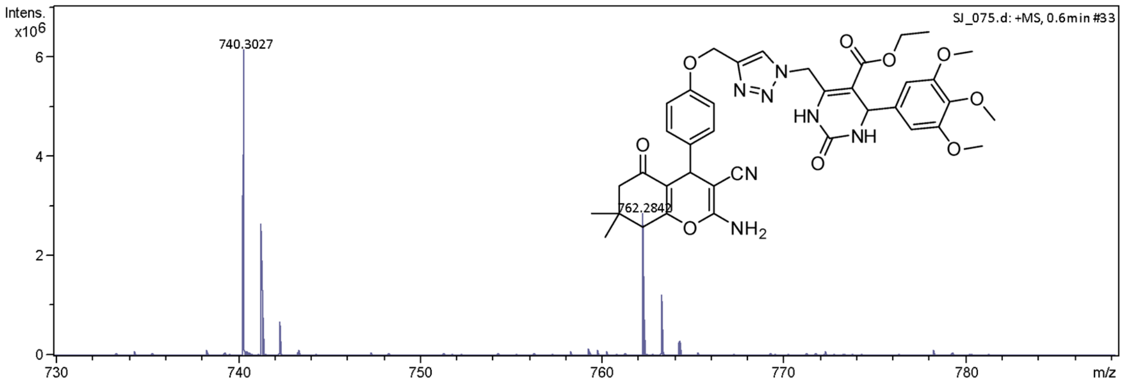

Meas. m/z	Ion Formula	m/z	err [ppm]	mSigma	rdb	e⁻ Conf	N-Rule
740.3027	$C_{38}H_{42}N_7O_9$	740.3039	1.6	9.0	21.5	even	ok
762.2842	$C_{38}H_{41}N_7NaO_9$	762.2858	2.1	9.0	21.5	even	ok

Figure S46 HRMS of Hybrid SJ075

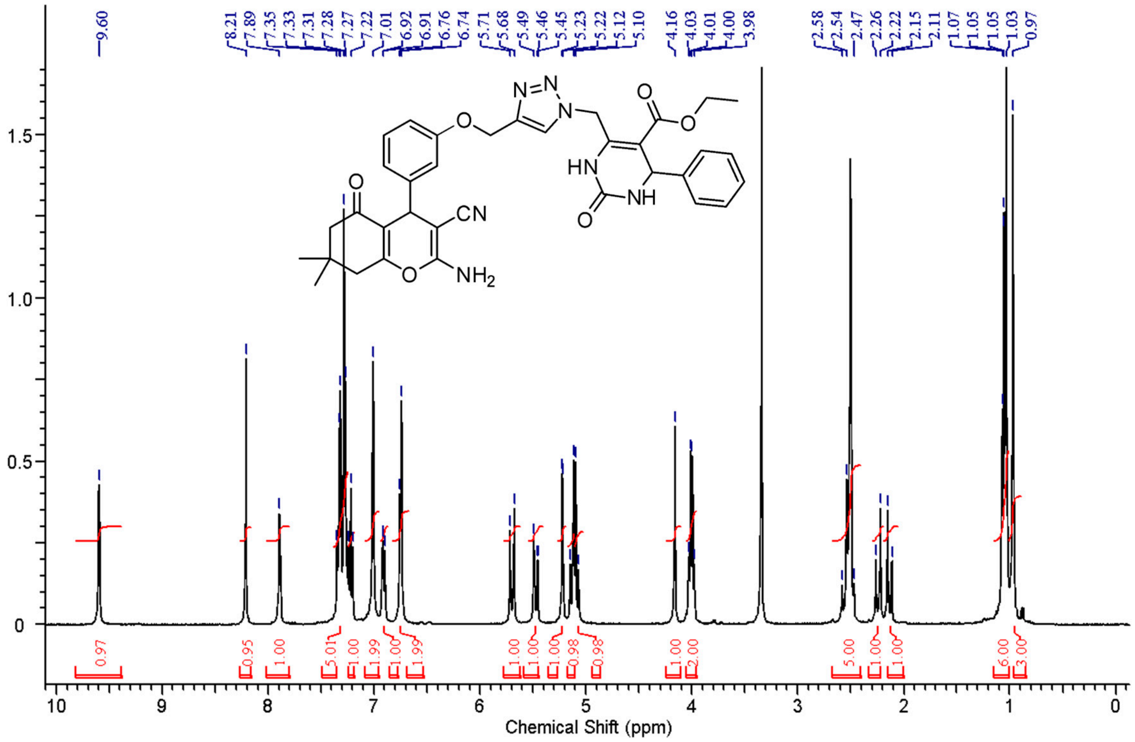

Figure 47: ^{1}H-NMR (400 MHz, DMSO-*d*6) of Hybrid Compound **SJ084**

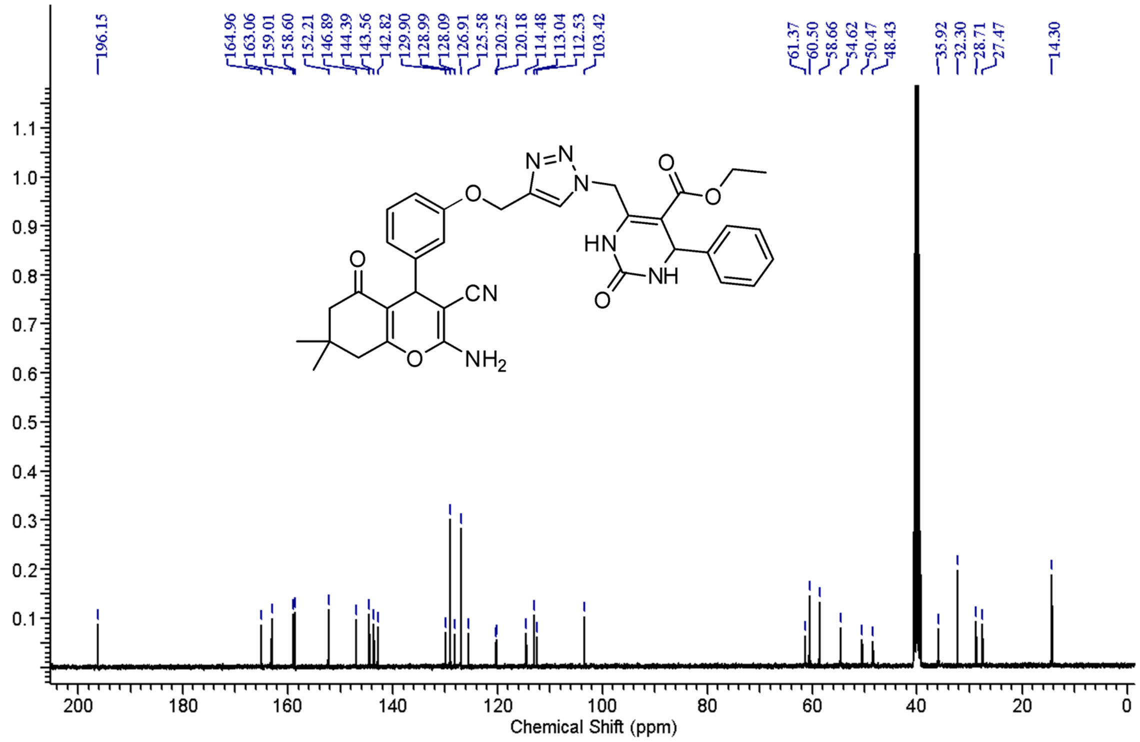

Figure 48: ^{13}C-NMR (100 MHz, DMSO-*d*6) of Hybrid Compound **SJ084**

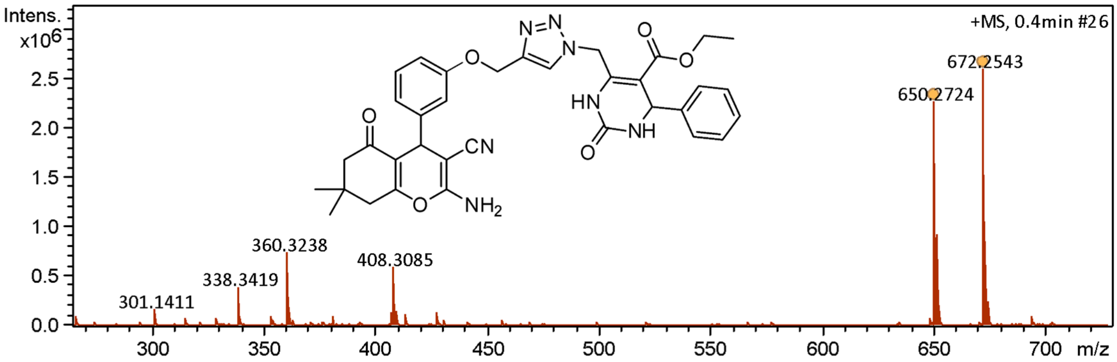

Meas. m/z	Ion Formula	m/z	err [ppm]	mSigma	rdb	e⁻ Conf	N-Rule
672.2543	$C_{35}H_{35}N_7NaO_6$	672.2541	-0.4	5.2	21.5	even	ok
650.2724	$C_{35}H_{36}N_7O_6$	650.2722	-0.4	3.1	21.5	even	ok

Figure S49: HRMS of Hybrid Compound **SJ084**

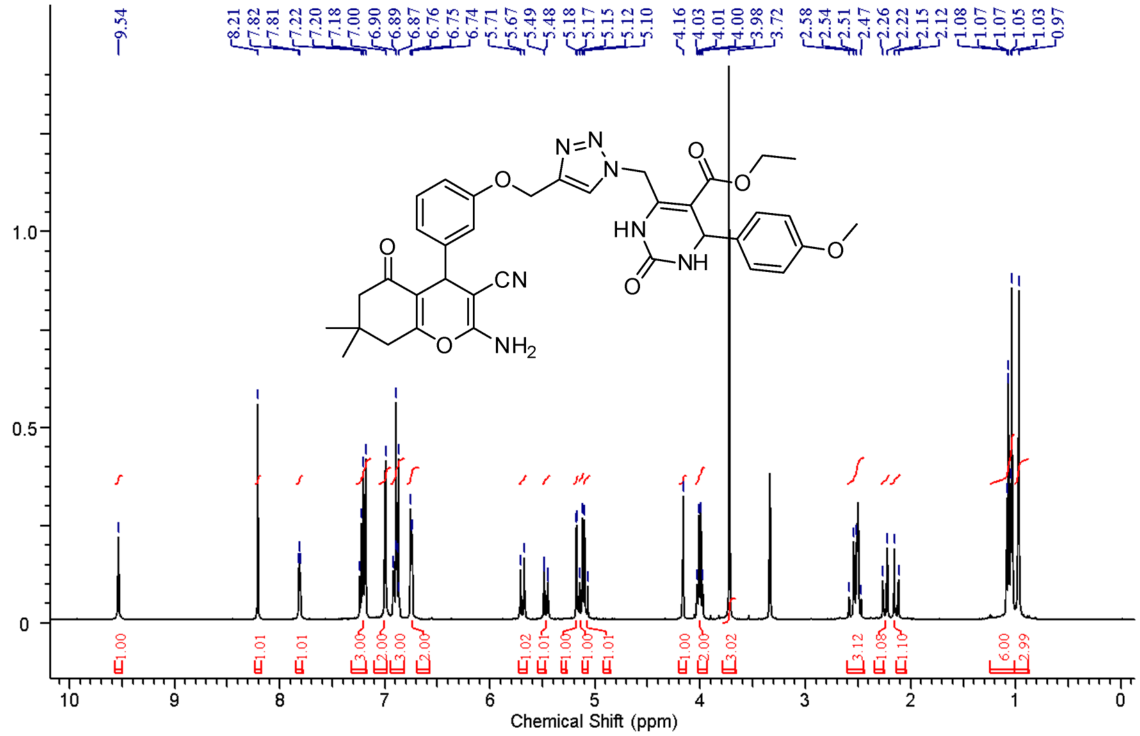

Figure S50: ^{1}H-NMR (400 MHz, DMSO-*d*6) of Hybrid Compound **SJ085**

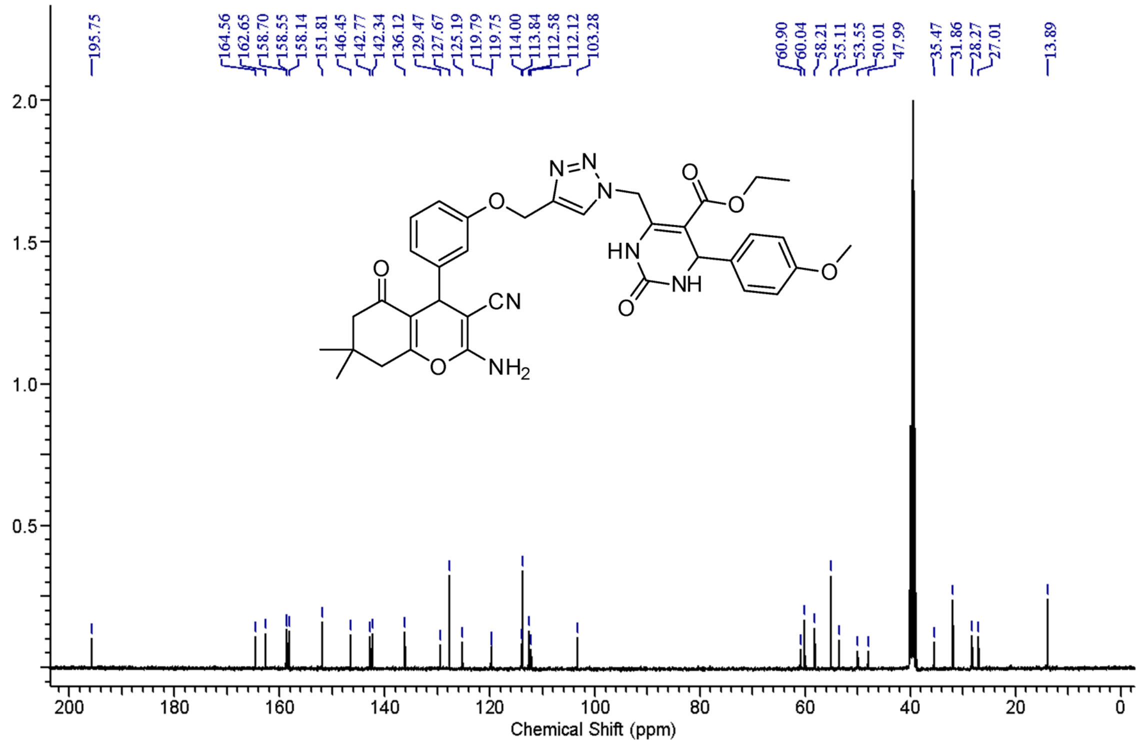

Figure S51: ^{13}C-NMR (100 MHz, DMSO-*d*6) of Hybrid Compound **SJ085**

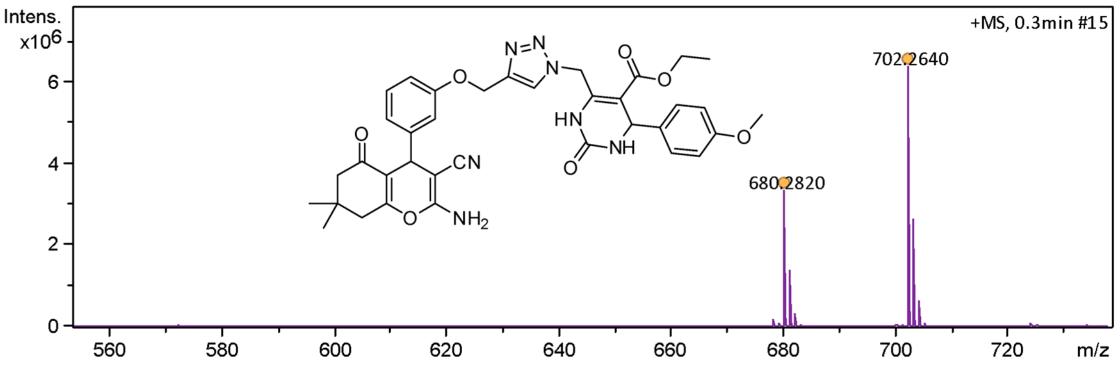

Meas. m/z	Ion Formula	m/z	err [ppm]	mSigma	rdb	e⁻ Conf	N-Rule
680.2820	$C_{36}H_{38}N_7O_7$	680.2827	1.1	5.6	21.5	even	ok
702.2640	$C_{36}H_{37}N_7NaO_7$	702.2647	0.9	5.6	21.5	even	ok

Figure S52: HRMS of Hybrid Compound **SJ085**

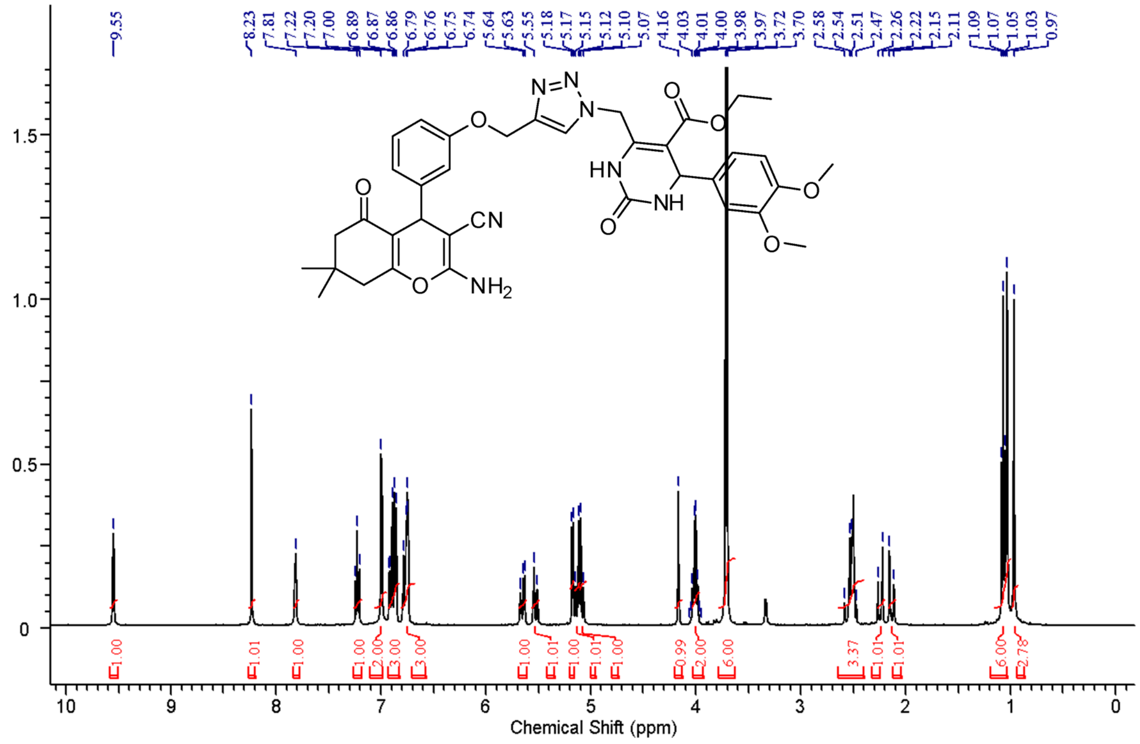

Figure S53: ^{1}H-NMR (400 MHz, DMSO-*d*6) of Hybrid Compound **SJ086**

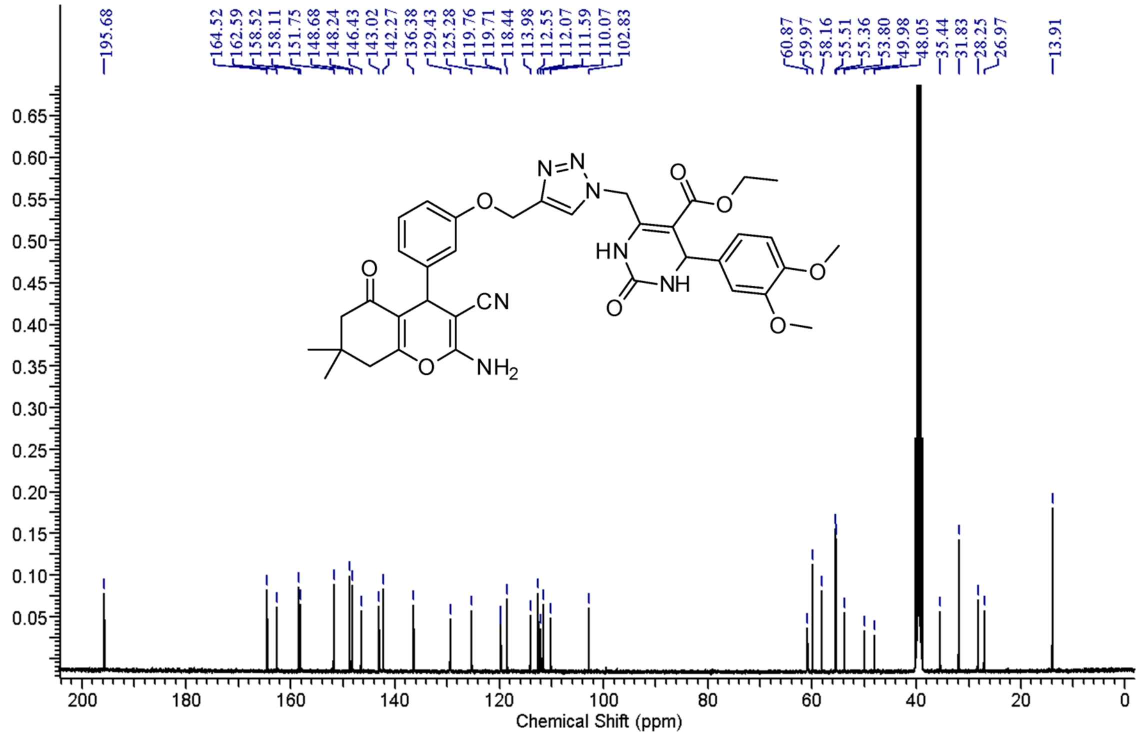

Figure S54: ^{13}C-NMR (100 MHz, DMSO-*d*6) of Hybrid Compound **SJ086**

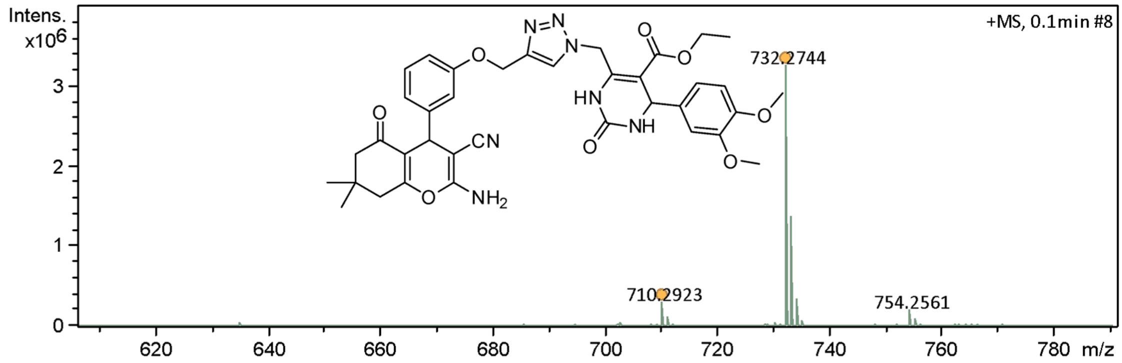

Meas. m/z	Ion Formula	m/z	err [ppm]	mSigma	rdb	e⁻ Conf	N-Rule
710.2923	$C_{37}H_{40}N7O_8$	710.2933	1.5	10.0	21.5	even	ok
732.2744	$C_{37}H_{39}N_7NaO_8$	732.2752	1.1	6.6	21.5	even	ok

Figure S55: HRMS of Hybrid Compound SJ086

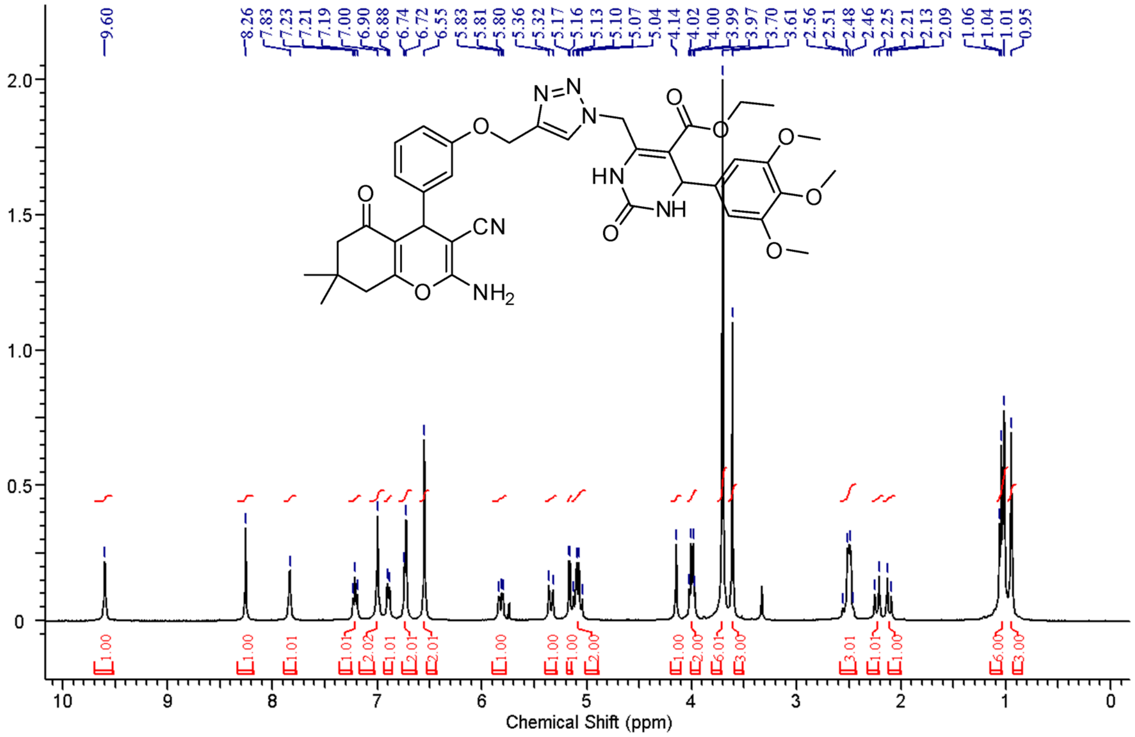

Figure S56: ^{1}H-NMR (400 MHz, DMSO-*d*6) of Hybrid Compound **SJ087**

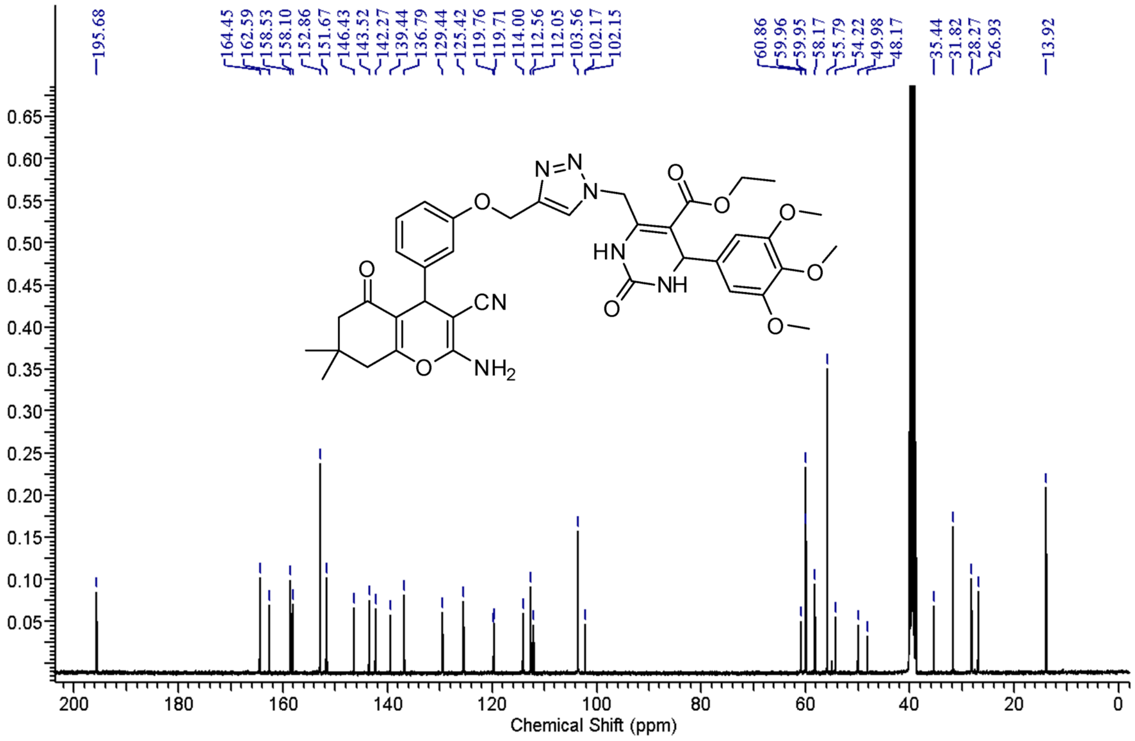

Figure S57: ^{13}C-NMR (100 MHz, DMSO-*d*6) of Hybrid Compound **SJ087**

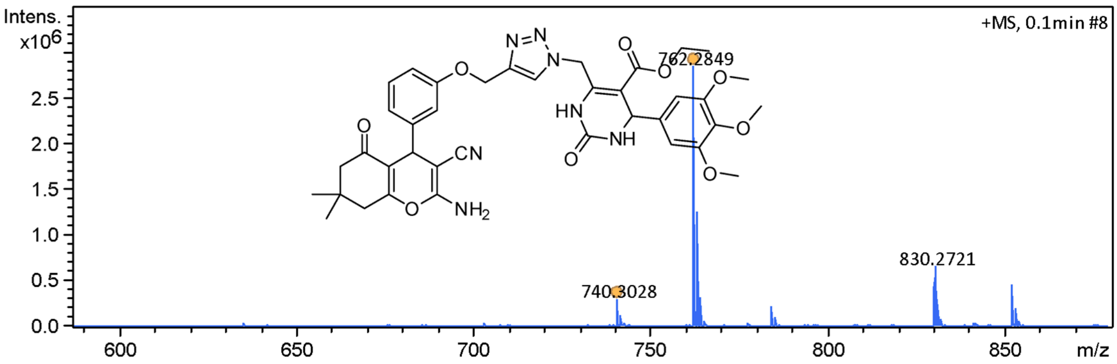

Meas. m/z	Ion Formula	m/z	err [ppm]	mSigma	rdb	e⁻ Conf	N-Rule
740.3028	$C_{38}H_{42}N7O_9$	740.3039	1.4	6.3	21.5	even	ok
762.2849	$C_{38}H_{41}N7NaO_9$	762.2858	1.1	2.6	21.5	even	ok

Figure S58: HRMS of Hybrid Compound SJ087

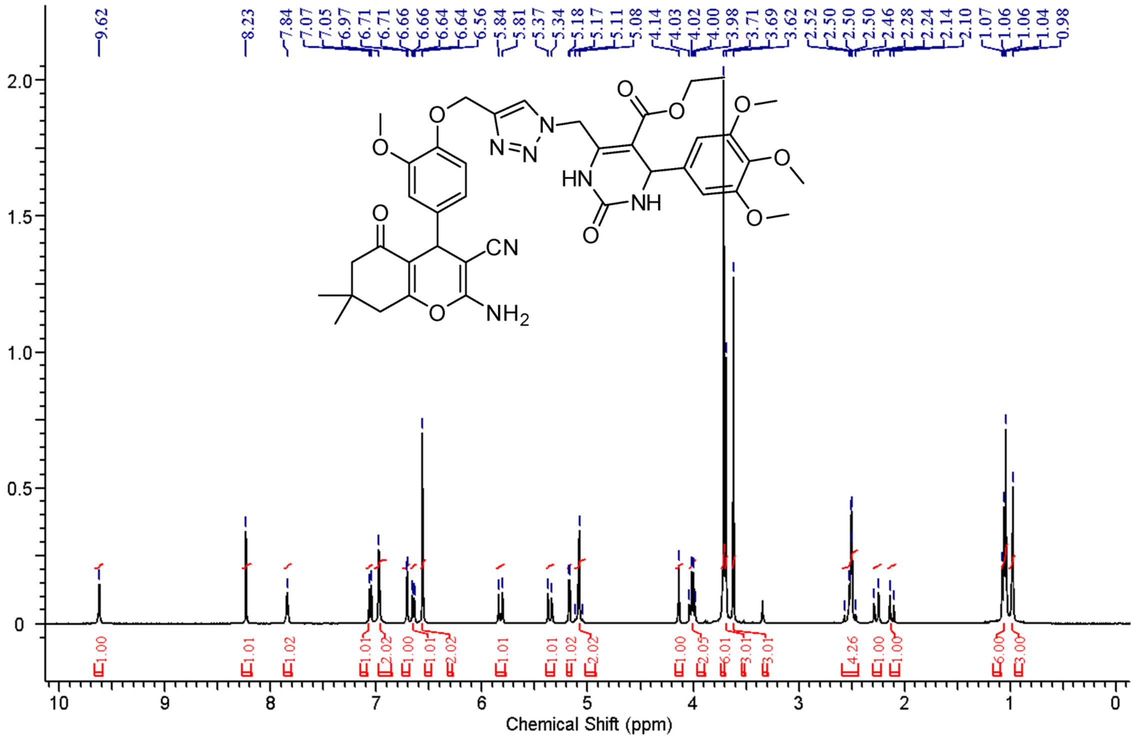

Figure S59: ^{1}H-NMR (400 MHz, DMSO-*d*6) of Hybrid Compound **SJ092**

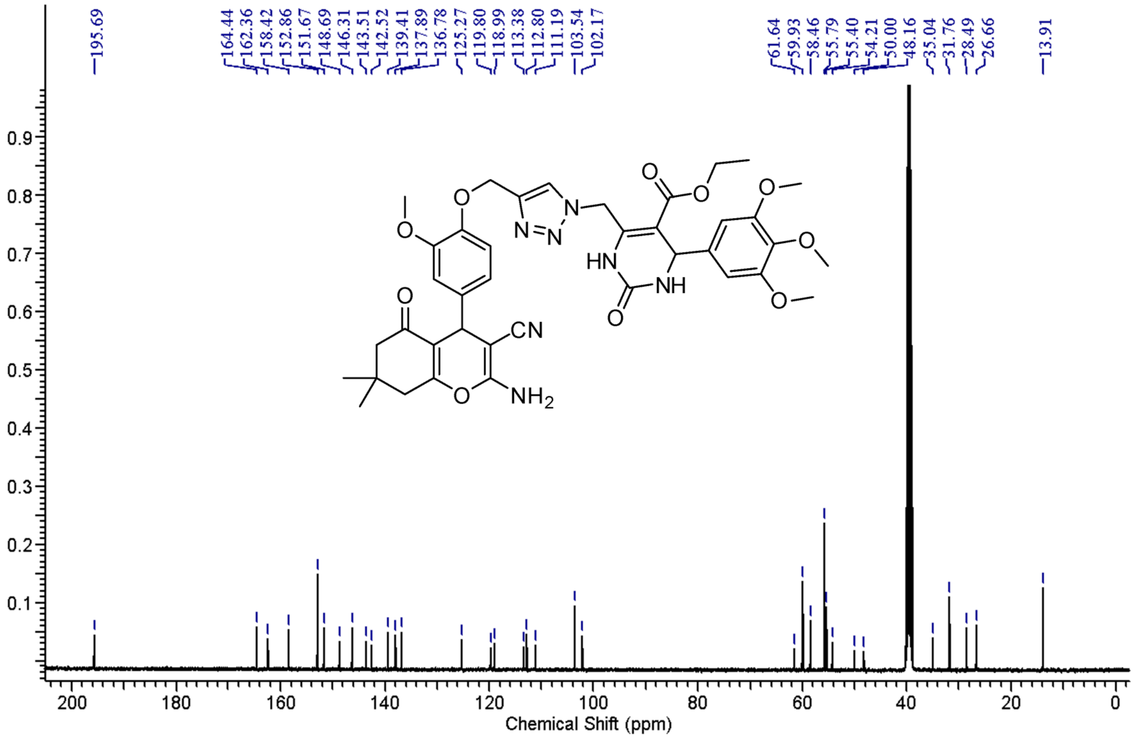

Figure S60: ^{13}C-NMR (100 MHz, DMSO-*d*6) of Hybrid Compound **SJ092**

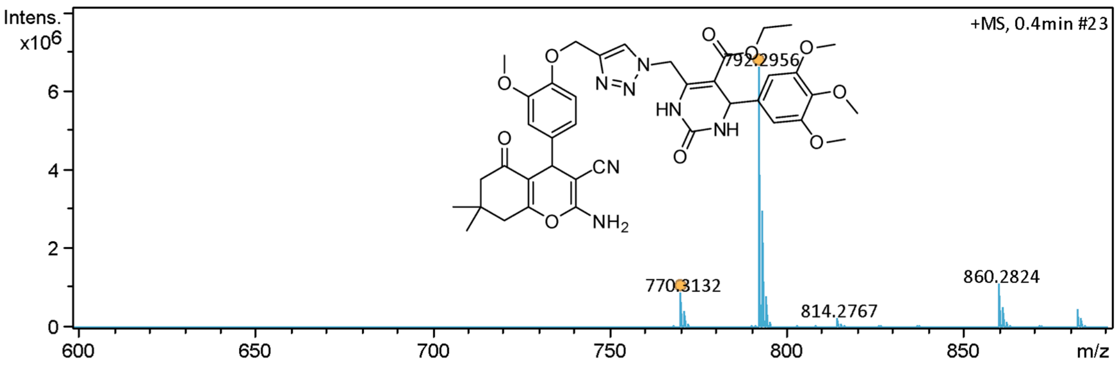

Meas. m/z	Ion Formula	m/z	err [ppm]	mSigma	rdb	e¯ Conf	N-Rule
792.2956	$C_{39}H_{43}N_7NaO_{10}$	792.2964	1.0	5.0	21.5	even	ok
770.3132	$C_{39}H_{44}N_7O_{10}$	770.3144	1.5	3.4	21.5	even	ok

Figure S61: HRMS of Hybrid Compound **SJ092**

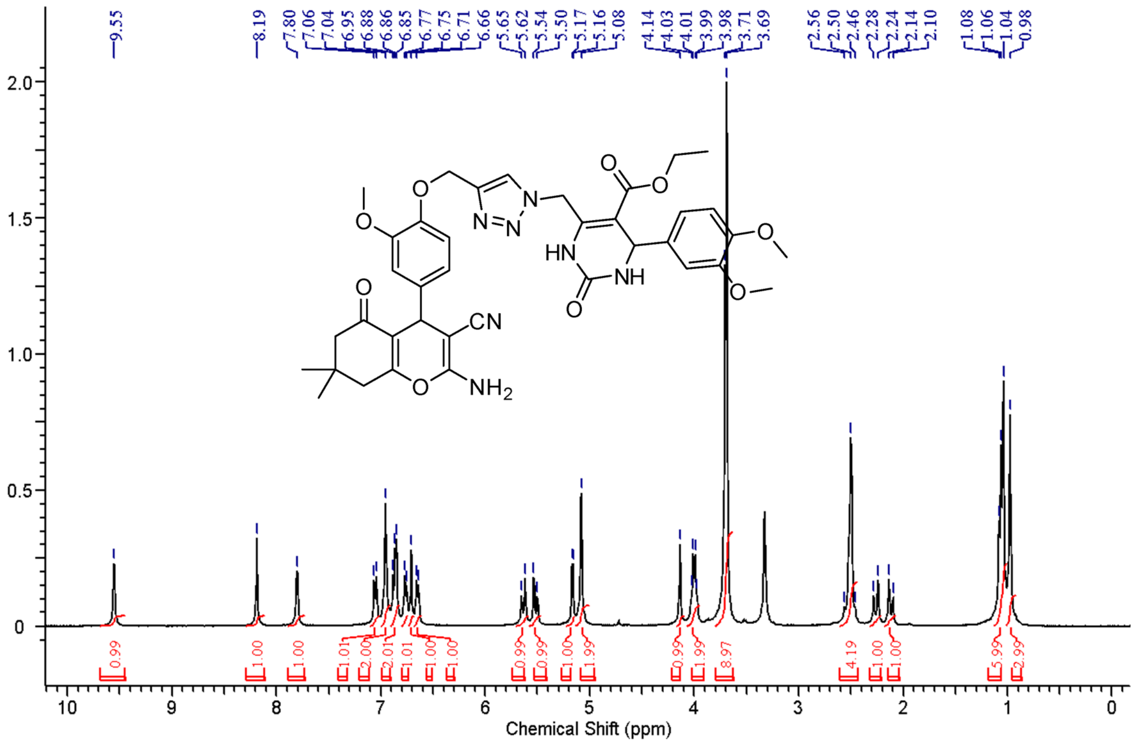

Figure S62: ^{1}H-NMR (400 MHz, DMSO-*d*6) of Hybrid Compound **SJ093**

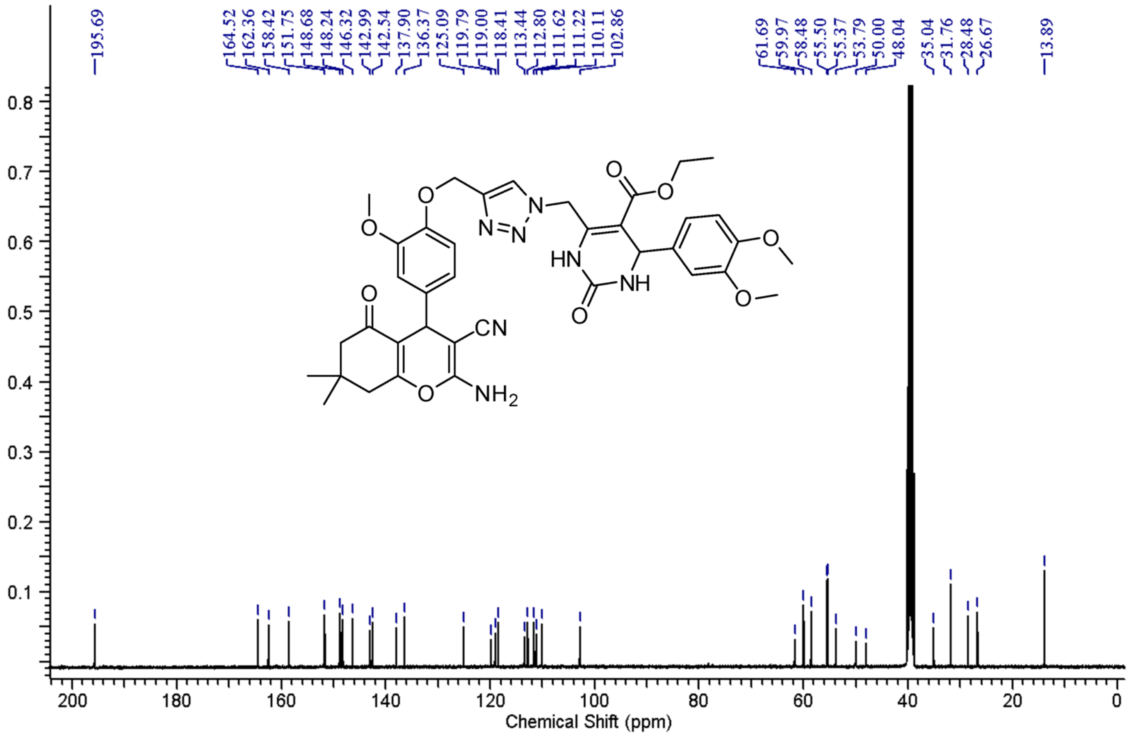

Figure S63: ^{13}C-NMR (100 MHz, DMSO-*d*6) of Hybrid Compound **SJ093**

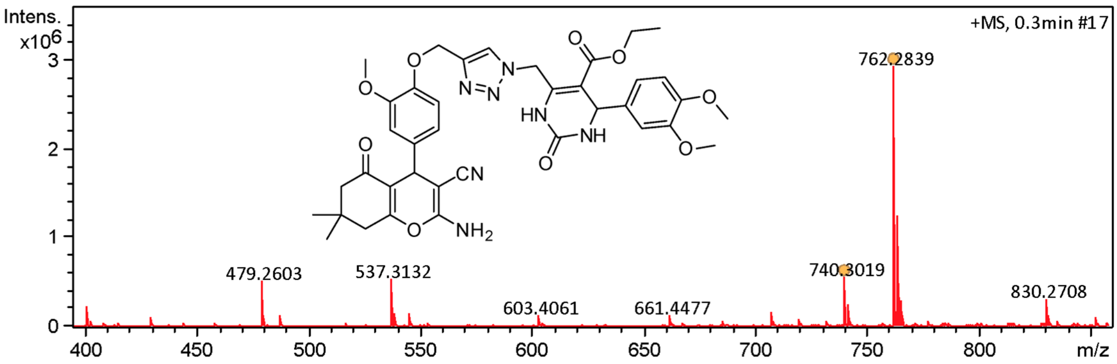

Meas. m/z	Ion Formula	m/z	err [ppm]	mSigma	rdb	e⁻ Conf	N-Rule
740.3019	$C_{38}H_{42}N_7O_9$	740.3039	2.7	8.3	21.5	even	ok
762.2839	$C_{38}H_{41}N_7NaO_9$	762.2858	2.5	8.5	21.5	even	ok

Figure S64: HRMS of Hybrid Compound SJ093

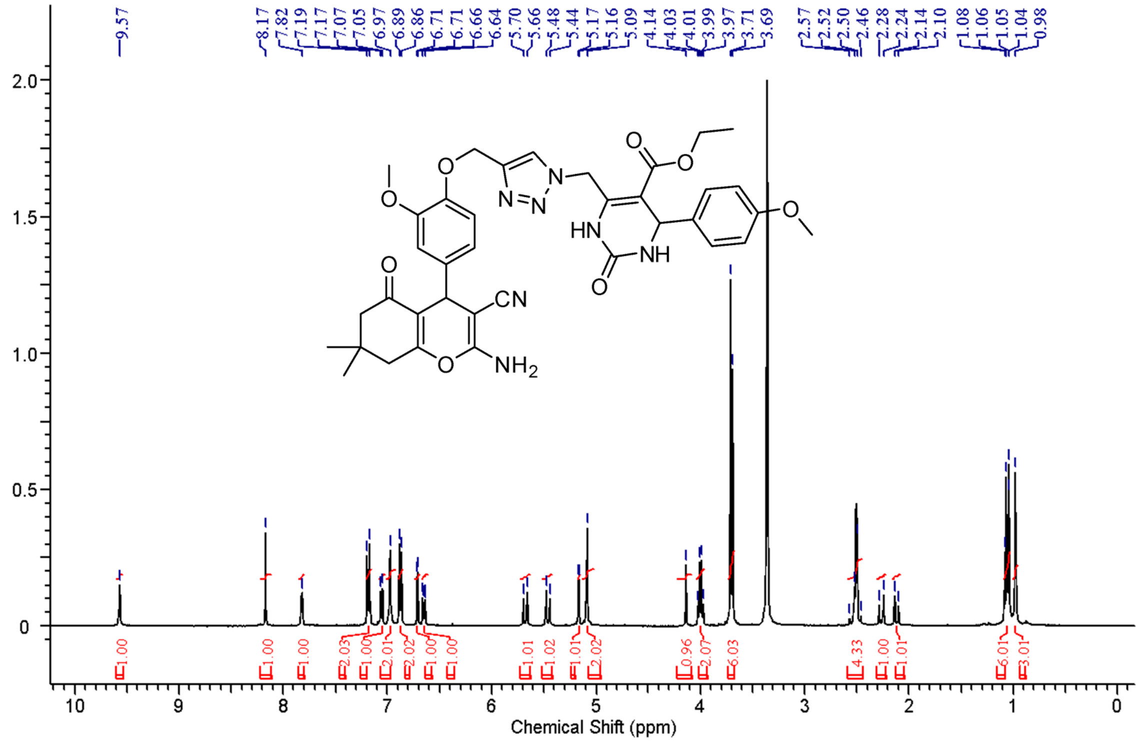

Figure S65: ^{1}H-NMR (400 MHz, DMSO-*d*6) of Hybrid Compound **SJ094**

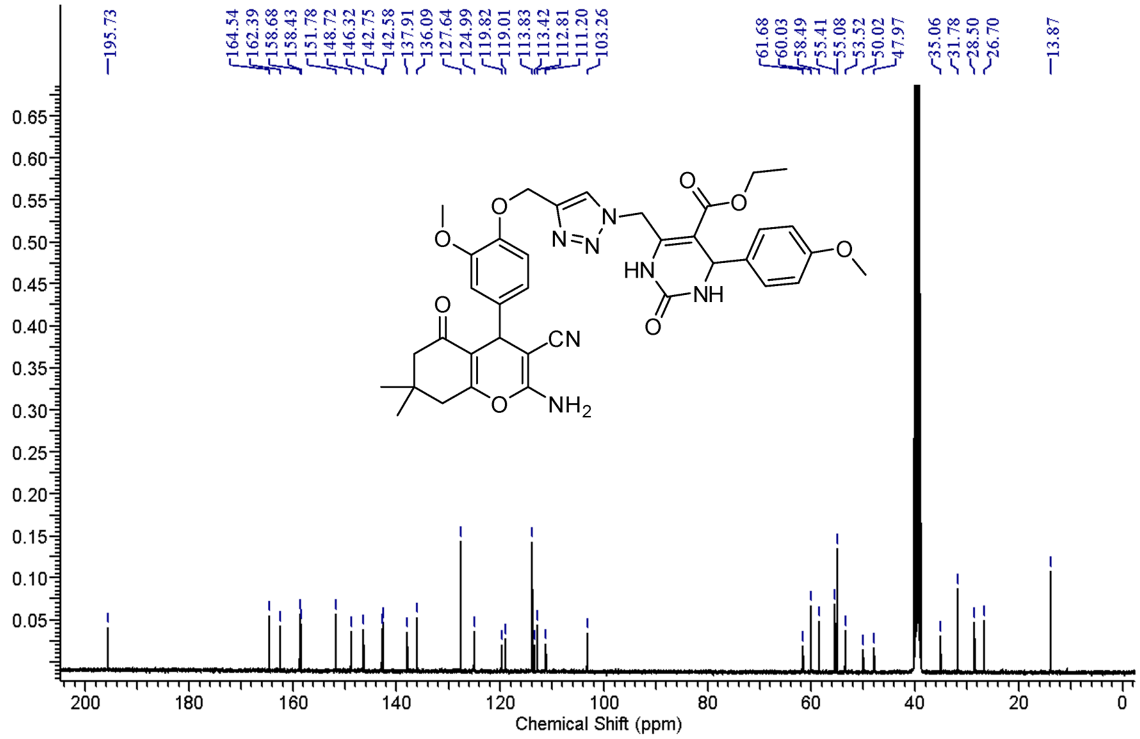

Figure S66: ^{13}C-NMR (100 MHz, DMSO-*d*6) of Hybrid Compound **SJ094**

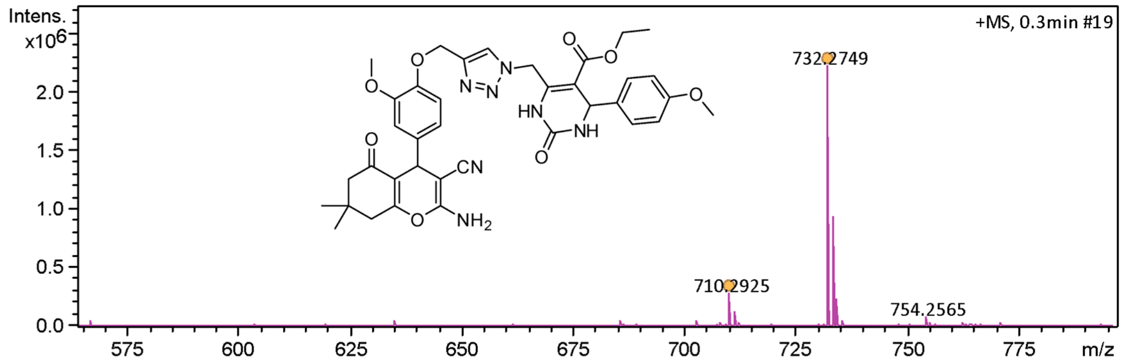

Meas. m/z	Ion Formula	m/z	err [ppm]	mSigma	rdb	e¯ Conf	N-Rule
732.2749	$C_{37}H_{39}N7NaO_8$	732.2752	0.5	6.9	21.5	even	ok
710.2925	$C_{37}H_{40}N_7O_8$	710.2933	1.1	10.0	21.5	even	ok

Figure S67: HRMS of Hybrid Compound **SJ094**
